# Biophysical modeling of *C. elegans* neurons: Single ion currents and whole-cell dynamics of AWC^on^ and RMD

**DOI:** 10.1371/journal.pone.0218738

**Published:** 2019-07-01

**Authors:** Martina Nicoletti, Alessandro Loppini, Letizia Chiodo, Viola Folli, Giancarlo Ruocco, Simonetta Filippi

**Affiliations:** 1 Department of Engineering, Campus Bio-Medico University, Rome, Italy; 2 Center for Life Nano Science CLNS@Sapienza, Istituto Italiano di Tecnologia - IIT, Rome, Italy; Georgia State University, UNITED STATES

## Abstract

*C. elegans* neuronal system constitutes the ideal framework for studying simple, yet realistic, neuronal activity, since the whole nervous system is fully characterized with respect to the exact number of neurons and the neuronal connections. Most recent efforts are devoted to investigate and clarify the signal processing and functional connectivity, which are at the basis of sensing mechanisms, signal transmission, and motor control. In this framework, a refined modelof whole neuron dynamics constitutes a key ingredient to describe the electrophysiological processes, both at thecellular and at the network scale. In this work, we present Hodgkin-Huxley-based models of ion channels dynamics black, built on data available both from *C. elegans* and from other organisms, expressing homologous channels. We combine these channel models to simulate the electrical activity oftwo among the most studied neurons in *C. elegans*, which display prototypical dynamics of neuronal activation, the chemosensory AWC^ON^ and the motor neuron RMD. Our model properly describes the regenerative responses of the two cells. We analyze in detail the role of ion currents, both in wild type and in *in silico* knockout neurons. Moreover, we specifically investigate the behavior of RMD, identifying a heterogeneous dynamical response which includes bistable regimes and sustained oscillations. We are able to assess the critical role of T-type calcium currents, carried by CCA-1 channels, and leakage currents in the regulation of RMD response. Overall, our results provide new insights in the activity of key *C. elegans* neurons. The developed mathematical framework constitute a basis for single-cell and neuronal networks analyses, opening new scenarios in the *in silico* modeling of *C. elegans* neuronal system.

## Introduction

*Caenorhabditis elegans* is a small, free-living nematode. Its short life cycle, small size (adults are 1.5 mm long), transparent body, limited anatomical complexity (∼1000 cells in adults), ease of genetic manipulation and low cost of maintenance made it a powerful tool for *in vivo* experiments. *C. elegans*, with its sequenced and conserved genome [1] and its simple but largely investigated nervous system [2–4], has become established as a standard model organism for a great variety of disciplines, ranging from developmental biology to neuroscience [5, 6].

This nematode is the only organism whose connectome has been fully reconstructed at the cellular level [7], representing a fundamental tool in neuroscience studies. Its nervous system, with only 302 neurons and ∼7000 synaptic connections, is still complex enough, at genetic, cellular and molecular level, to generate and control complex neuronal functions such those related to sensory information processing and motor control. It has to be underlined that a full network investigation requires, in principle, the complete knowledge not only of the single neuron dynamics, but also of strength and polarity of all connections [4], which are currently not completely characterized. Despite this partial lack of information, still the nematode nervous system constitutes the ideal framework for understanding sensory integration processes, information flow and output control mechanisms.

A proper understanding of the neuronal transmission and electrical response, at the molecular level, is based on the study of the membrane ion channels activity. A vast, but somehow sparse, literature addresses the role of specific ion channels in the functioning of various cells in *C. elegans*, from neurons, including sensory [8–10], inter- [11, 12] and motor neurons [13, 14], to cells in afferent muscles [15–20]. However, it is still missing a complete electrophysiological description and characterization of whole-cell ion currents mostly because of experimental issues. Indeed, electrophysiological measurements are particularly challenging due to cells accesibility (the average soma radius is ∼ 1 μm), and complications in preserving worm life and cells functionality after dissection. Notably, various computational and mathematical models, based on realistic biological parameters, have been developed to describe the whole-cell spatiotemporal activity of a *C. elegans* neurons. However, they are mostly based on simplified ion currents description, or they focus only on the calcium dynamics [10, 21–23], having therefore a limited applicability. To date, there is no report of a comprehensive electrophysiological model taking into account the contribution of all the main ion currents involved in the activity of *C. elegans* neurons.

In this paper, we model the electrical dynamics at the single neuron level, to provide a tool useful in investigating neuron functioning, in driving targeted experiments on *C. elegans* neurobiology and in developing *in silico* experiments on both single neurons and networks.

To develop our model, we rely on a Hodgkin-Huxley formulation [24] to describe the main channels participating in the neuronal activity, according to genetic expression, electrophysiological data [25–28] and open access database (Wormbase: https://www.wormbase.org). Specifically, we use available experimental data [11, 12, 15, 25, 26, 28–44] to build 14 detailed ion channel models (Table 1) with specific ion selectivity: voltage-gated potassium channels (KV), voltage-gated calcium channels (CaV), and calcium-regulated potassium channels (KCa).

**Table 1:** List of modeled ion channels. The modeled *C. elegans* ion channels (second column), grouped on the basis of ion selectivity (first column) are classified according to vertebrate homologues (third column) and corresponding nematode genes (fourth column). The fifth and the sixth columns report the gene expression in AWC^ON^ and RMD. The seventh column reports the references to experimental data used to model each channel type and the relative organism. The symbols denote the specific patch clamp recording conditions: • on dissected worm cultured myocytes [25], body wall muscles [15] or pharynx [36], *, ∘ and ⋄ respectively on *Xenopus* oocytes, HEK cells, and CHO cells expressing the desired genes. In the text, the genes encoding for the modeled channels and the corresponding channel proteins are reported in italic and capital letters, respectively (e.g *egl-19* genes encode for EGL-19 channels). Further, we denote the current associated to a specific class of channels with the channel protein name, omitting the dash between the gene family name and the number (e.g. EGL19 are the currents carried by EGL-19 channels). When two or more genes encode for a channel type, we indicate the channels and the currents with the gene family name in capital letters (e.g. *kcnl-1/4*, KCNL).

*Ion*	*Channel type*	*Vertebrate gene family*	*C. elegans gene*	*AWC^ON^*	*RMD*	*Experimental data*
**K^+^**	Kv1,*shaker*	*kcna*	*shk-1*	✓	✓	[25]: *C*.*elegans*^•^ [29]: Human*
	Kv3,*shaw*	*kcnc*	*egl-36*	-	✓	[30]: *C*.elegans*
	Kv4,*shal*	*kcnd*	*shl-1*	✓	✓	[25]: *C*.*elegans*^•^
	Kvs-1		*kvs-1*	✓	-	[31]: *C*.*elegans*^⋄^
	Kv7	*kcnq*	*kqt-3*	✓	-	[26]: *C*.elegans*
	Kv10, *eag*	*kcnh*	*egl-2*	✓	-	[28]: *C*.elegans* [32]: Drosophila*
	Kir	*kcnj*	*irk1-3*	✓	✓	[33]: *C*.elegans* [34]: *Rat* [35]: *Aplysia californica*
**Ca^2+^**	CaV1, L-type	*cacna*	*egl-19*	✓	✓	[15, 36]: *C*.*elegans*^•^
	CaV2, P/Q-type	*canca*	*unc-2*	✓	✓	[37]: *Rat*^∘^ [38]: *Rat* [39]: *Mammalian*^∘^
	CaV3, T-type	*canca*	*cca-1*	✓	✓	[40]: Drosophila*
**K^+^**	KCa1.1, BK	*slo1*	*slo-1*	✓	✓	[41]: *C*.elegans*
	BK	*slo2, slack*	*slo-2*	✓	✓	[42]: *C*.elegans*
	KCa2, SK	*kcnn*	*kcnl-1/4*	✓	✓	[43] *No published patch clamp data*
**Na^+^**	NALCN	*nalcn*	*nca-1/2*	✓	✓	[11, 12, 44] *No published patch clamp data*

The potassium channels are ubiquitous and widely expressed in *C. elegans*, with 72 genes encoding three classes of channels: 2TM, 4TM and 6TM [45]. Potassium channels are particularly important both for shaping electrical signals in neurons and for setting the resting potential in almost all excitable cells. In *C. elegans*, both voltage-gated and calcium-regulated potassium channels can be found. The voltage-gated channels regulate the electrical activity of neurons and muscles by setting the membrane resting potential [14], shaping the action potentials and regulating the firing rate in muscles [17, 19]. In this work, we modeled 7 voltage-gated potassium channels: SHL-1, KVS-1, SHK-1, IRK1-3, KQT-3, EGL-36, EGL-2.

The calcium-regulated potassium channels (KCa) directly tune the cell electrical activity upon intracellular calcium modulation and are important regulators of neuronal activity in this nematode. They are classified depending on the value of single channel conductance: big conductance (BK, ∼100 pS), intermediate (∼50 pS), and small conductance (SK, ∼10 pS) channels [46–49]. Among them, the most relevant for neuronal activity in *C. elegans* are BK and SK channels. Some important differences exist between BK and SK channels: the former are modulated both by membrane voltage and intracellular calcium concentration, while the latter are voltage-independent and thus modulated solely by calcium. BK and SK channels also differ in their sensitivity to calcium changes. SK channels are usually active at physiological calcium concentrations and are very sensitive to small changes, caused by a variety of sources such as CaV, Na^+^-Ca^+^ exchangers, and Ca^2+^-ATPases. BK channels are normally activated at very high calcium concentrations, reached only in the proximity of voltage-gated calcium channels. Indeed, they colocalize with CaV in nanodomains forming complexes with one or more calcium channels [50–54]. In this work, we modeled three calcium-regulated channels: two BK (SLO-1, SLO-2) and one SK channel (KCNL-1/4). To properly take into account the differences between BK and SK, we modeled the intracellular calcium changes by using two different approaches [51, 55].

The role of voltage-gated calcium channels (CaV) is essential in *C. elegans* because it lacks the Na^+^ channels that generate action potentials in vertebrates [56]. Indeed, it was shown that *C. elegans* neurons could generate active responses such as regenerative (sensory neurons [57]) and plateau potentials (RMD motor neurons [13, 58]), and only limited action potentials (AWA neurons [10]). In this work we modeled the three main classes of voltage-gated calcium currents, T-type, L-type and P/Q-type, which have been measured in both *C. elegans* neurons and muscles. [15, 36, 37, 40]. L-type currents are carried by EGL-19 channels, while P/Q and T-type are carried by UNC-2 and CCA-1 channels,respectively [56].

We use modeled ion currents to build a well constrained biophysical model of neuronal electrical activity, able to capture the whole-cell current dynamics and to be used to investigate specific ion current contributions to the overall response upon stimulation. We validate the model by reproducing the behavior of two of the most studied neurons of this nematode: the sensory neuron AWC^ON^ and the motor neuron RMD, representative of two fundamental neuronal classes in *C. elegans*, and showing both regenerative and plateau potentials. Our model is validated against available patch-clamp data [13, 59].

AWC neurons are amphid sensory neurons responsible for the chemotaxis towards volatile odorants [60]. AWC exhibits a left-right asymmetry related to the stochastic expression of the GPCRs STR-2 in AWC^ON^ and SRX-3 in AWC^OFF^ [61, 62]. AWC is also involved in temperature, light and electric fields sensation [63–65]. The RMD neurons are ring motor neurons responsible for spontaneous foraging movements and head withdrawal reflex, that are activated by the stimulation of the mechanosensory neurons OLQ and IL1. RMD neurons can exhibit regenerative plateau potentials, triggered by the sequential activation of calcium channels, with a bistable resting potential [13].

This work provides, for the first time to the best of the authors’ knowledge, a comprehensive and detailed *in silico* investigation of the overall neuronal dynamics for this nematode. The detailed biophysical model presented in this work has the potential to accurately describe the electrical activity underlying neuron functions, to identify key molecular mechanisms in signal generation and transmission and to integrate partial and missing information in other models, focusing on specific intracellular variables. In particular, the improved description of electrical activity, shaping the calcium fluxes, can contribute to develop most refined calcium dynamics models. This work gives new perspectives in quantitative modeling of electrical activity in *C. elegans*, to be used also in a predictive scenario, both at the single cell scale and in the case of neuronal networks.

## Methods

In this section, we describe the mathematical model, including voltage-gated and calcium-regulated ion currents, intracellular calcium modeling, and numerical integration and fitting procedure. Our biophysical model is based on a Hodgkin-Huxley type formulation. Ionic currents are modeled based on experimental data on *C. elegans*, when available, or on other organisms expressing homologous channels (see Table 1), and finally combined to reproduce whole-cell electrical activity. All modeled channels are listed in Table 1, associated with their expression profile in the two neurons here analyzed, namely AWC^ON^ and RMD.

### Channel modeling

Modeled currents are potassium voltage-gated currents (SHL1, KVS1, SHK1, IRK, KQT3, EGL36, EGL2), calcium voltage-gated currents (EGL19, UNC2, CCA1), calcium-regulated potassium currents carried by big conductance channels (SLO1/SLO2 in combination with EGL19/UNC2) and small conductance channels (KCNL), sodium passive currents (NCA) and non-specific passive currents (LEAK). The majority of currents included in the model (voltage-gated potassium and calcium) are described with the standard mathematical formulation [24]:

$$\begin{array}{c} I_{x}=g_{x}\cdot \left(V-V_{x}\right)={\bar{g}}_{x}\cdot m_{x}^{p}\cdot h_{x}^{q}\cdot \left(V-V_{x}\right)\;, \end{array}$$ (1)

where *x* denotes a generic ion channel, *V*_*x*_ represents the reversal potential for the current passing through the channel *x*, ${\bar{g}}_{x}$ is the whole-cell maximal conductance associated with *x*, *m*_*x*_ and *h*_*x*_ denote activation and inactivation gating variables and the parameters *p* and *q* are assumed both equal to 1, unless stated otherwise.

Activation and inactivation dynamics of each current follow the first-order differential equations

$$\begin{array}{c} \frac{dm_{x}}{dt}=\frac{m_{x,\infty }\left(V\right)-m_{x}}{\tau_{m_{x}}\left(V\right)} \end{array}$$ (2)

$$\begin{array}{c} \frac{dh_{x}}{dt}=\frac{h_{x,\infty }\left(V\right)-h_{x}}{\tau_{h_{x}}\left(V\right)}\; \end{array}$$ (3)

where *m*_*x*,∞_(*V*) (*h*_*x*,∞_(*V*)) denotes the steady-state activation (inactivation) function, and $\tau_{m_{x}}\left(V\right)$ ($\tau_{h_{x}}\left(V\right)$) represents the activation (inactivation) voltage-dependent time constant.

The voltage-dependence of steady-states and time constants are modeled with Boltzmann-like functions, unless stated otherwise:

$$\begin{array}{c} m_{x,\infty }\left(V\right)=\frac{1}{1-e^{\frac{-(V-V_{0.5})}{k_{a}}}} \end{array}$$ (4)

$$\begin{array}{c} h_{x,\infty }\left(V\right)=\frac{1}{1+e^{\frac{\left(V-V_{0.5}\right)}{k_{i}}}} \end{array}$$ (5)

$$\begin{array}{c} \tau_{m_{x}}\left(V\right)=\tau_{h_{x}}\left(V\right)=\frac{a}{1+e^{\frac{\left(V-b\right)}{c}}}+d\;. \end{array}$$ (6)

Sodium (NCA) and non-specific leak currents (LEAK) are modeled as standard passive ohmic currents $I_{x}={\bar{g}}_{x}\cdot \left(V-V_{x}\right)$. A complete list of model equations for currents, steady-state activation and inactivation curves and time constant functions is given in Supplemental material (Eqs A-C in S1 File).

The study of calcium-regulated BK and SK channels, with currents regulated by both ligands and voltage, requires the use of specific models, reported in the following paragraphs.

Relying on the general formulation for ion currents described above, whole-cell membrane voltage dynamics is described by:

$$\begin{array}{c} C\frac{dV}{dt}=-I_{\text{ion}}+I_{\text{ext}}\;. \end{array}$$ (7)

*I*_ext_ is the external applied current and *I*_ion_ contains all the considered ionic currents:

$$\begin{array}{c} \begin{array}{cc} I_{\text{ion}}= & {\left(I_{\text{SHL1}}+I_{\text{KVS1}}+I_{\text{SHK1}}+I_{\text{IRK}}+I_{\text{KQT3}}+I_{\text{EGL36}}+I_{\text{EGL2}}\right)}_{\text{KV}}\\ & +{\left(I_{\text{EGL19}}+I_{\text{UNC2}}+I_{\text{CCA1}}\right)}_{\text{CaV}}\\ & +{\left(I_{\text{SLO1/EGL19}}+I_{\text{SLO1/UNC2}}+I_{\text{SLO2/EGL19}}+I_{\text{SLO2/UNC2}}+I_{\text{KCNL}}\right)}_{\text{KCa}}\\ & +I_{\text{NCA}}+I_{\text{LEAK}}\;. \end{array} \end{array}$$ (8)

#### BK channels modeling

We adopt the model of isolated BK channels (uncoupled from CaV channels) to fit calcium and voltage sensitivity based on data for *C. elegans* SLO-1 [41] and SLO-2 [42] channels. Fitted parameters are then used to model the complete BK-CaV complex dynamics, as detailed below. For the sake of clarity, we report the complete description of the model [51]. The equation describing the isolated BK open state as function of applied voltage and calcium concentration is:

$$\begin{array}{c} \frac{dm_{\text{BKs}}}{dt}=\frac{m_{\text{BKs},\infty }\left(V,Ca\right)-m_{\text{BKs}}}{\tau_{m_{\text{BKs}}}\left(V,Ca\right)}, \end{array}$$ (9)

where *m*_BKs,∞_ = *k*^+^/(*k*^+^+ *k*^−^), $\tau_{m_{\text{BKs}}}=1/\left(k^{+}+k^{-}\right)$, and *k*^−^ and *k*^+^ are rate constants dependent both on calcium and voltage, expressed as:

$$\begin{array}{cc} k^{-} & =w^{-}\left(V\right)f^{-}\left(Ca\right) \end{array}$$ (10)

$$\begin{array}{cc} k^{+} & =w^{+}\left(V\right)f^{+}\left(Ca\right) \end{array}$$ (11)

with

$$\begin{array}{cc} w^{-}\left(V\right) & =w_{0}^{-}e^{-w_{yx}V} \end{array}$$ (12)

$$\begin{array}{cc} w^{+}\left(V\right) & =w_{0}^{+}e^{-w_{xy}V} \end{array}$$ (13)

$$\begin{array}{cc} f^{-}\left(Ca\right) & =\frac{1}{1+{\left(\frac{Ca}{K_{yx}}\right)}^{n_{yx}}} \end{array}$$ (14)

$$\begin{array}{cc} f^{+}\left(Ca\right) & =\frac{1}{1+{\left(\frac{K_{xy}}{Ca}\right)}^{n_{xy}}}\;. \end{array}$$ (15)

The steady-state activation *m*_BKs,∞_ is a standard Boltzmann function, with the half activation voltage and the slope written as functions of the intracellular calcium concentration and of the eight parameters $w_{0}^{+}$, $w_{0}^{-}$, *w*_*yx*_, *w*_*xy*_, *n*_*yx*_, *n*_*xy*_, *K*_*yx*_, and *K*_*xy*_:

$$\begin{array}{cc} & m_{\text{BKs},\infty }\left(V,Ca\right)=\frac{1}{1+e^{-\frac{V-V_{a}^{0.5}}{k_{a}}}} \end{array}$$ (16)

$$\begin{array}{cc} & V_{a}^{0.5}=k_{a}[\log(\frac{w_{0}^{-}}{w_{0}^{+}})+\log(1+{\left(\frac{K_{xy}}{Ca}\right)}^{n_{xy}})-\log(1+{\left(\frac{Ca}{K_{yx}}\right)}^{n_{yx}})] \end{array}$$ (17)

$$\begin{array}{cc} & k_{a}=\frac{1}{w_{yx}-w_{xy}}\;, \end{array}$$ (18)

where *K*_*yx*_ (*K*_*xy*_) is linked to calcium affinity for channel closing (opening) transition, *w*_*yx*_ (*w*_*xy*_) regulates voltage sensitivity of closing (opening) transition, $w_{0}^{-}$ ($w_{0}^{+}$) denotes the closing (opening) transition rate at 0 mV, and *n*_*yx*_ (*n*_*xy*_) represents the Hill coefficient of the closing (opening) transition. The activation time constant is a function of the same parameters,

$$\begin{array}{c} \tau_{m_{\text{BKs}}}\left(V,Ca\right)=\frac{e^{w_{xyV}}}{w_{0}^{+}}(1+{\left(\frac{K_{xy}}{Ca}\right)}^{n_{xy}})\frac{1}{1+e^{-\frac{V-V_{a}^{0.5}}{k_{a}}}}\;. \end{array}$$ (19)

Isolated SLO1 and SLO2 currents are given by:

$$\begin{array}{c} I_{\text{BKs}}={\bar{g}}_{\text{BK}}\cdot m_{\text{BKs}}\cdot \left(V-V_{K}\right)\;. \end{array}$$ (20)

The activation of BK channels is dependent on the state of nearby calcium channels [51, 52]. When CaV is closed, the opening probability for the corresponding BK channel is almost zero, since the calcium concentration inside the nanodomain is too low to cause a robust activation. The coupling between BK and CaV channel is modeled through the whole BK-CaV complex, for 1:1 stoichiometry [51], with the following equations for the opening probability and activation time constants:

$$\begin{array}{ccc} m_{\text{BK},\infty }\left(V,Ca\right) & = & \frac{m_{\text{CaV}}k_{o}^{+}\left(\alpha +\beta +k_{c}^{-}\right)}{\left(k_{o}^{+}+k_{o}^{-}\right)\left(k_{c}^{-}+\alpha \right)+\beta k_{c}^{-}} \end{array}$$ (21)

$$\begin{array}{ccc} \tau_{m_{\text{BK}}}\left(V,Ca\right) & = & \frac{\alpha +\beta +k_{c}^{-}}{\left(k_{o}^{+}+k_{o}^{-}\right)\left(k_{c}^{-}+\alpha \right)+\beta k_{c}^{-}} \end{array}$$ (22)

where *m*_CaV_ is the activation variable of the voltage-gated Ca^2+^ channel, $\alpha =\frac{m_{\text{CaV},\infty }}{\tau_{m_{\text{CaV}}}}$ and $\beta =\tau_{m_{\text{CaV}}}^{-1}-\alpha$. Transition rates $k_{o}^{+/-}$ and $k_{c}^{-}$ are calculated according to Eqs 10–15, by considering calcium inflow through open (*o*) CaV or calcium baseline in case of closed (*c*) CaV, respectively (see Intracellular calcium modeling section). The total current flowing across BK channels within the BK-CaV complex is given by:

$$\begin{array}{c} I_{\text{BK}}={\bar{g}}_{\text{BK}}\cdot m_{\text{BK}}\cdot h_{\text{CaV}}\cdot \left(V-V_{K}\right)\;. \end{array}$$ (23)

It is important to stress that the BK-CaV complex model (Eqs 21 and 22) depends on parameters extracted from the isolated BK channel description. Therefore, it is necessary to consider also the isolated BK channel to give a comprehensive description of the complex, in line with similar modeling studies [51]. Also, we remark that data on voltage and calcium sensitivity are available only for the isolated BK. In our simulations, we use Eqs 9, 16, 19 and 20 to reproduce the isolated BK current at fixed calcium concentrations, while we use Eqs 21, 22, 23 and 9 (the last one has to be considered by replacing *m*_BKs_ with *m*_BK_) to model BK current within the complex in the whole-cell model. In this case, calcium feedback depends on the single CaV channel current and nanoscale diffusion, as explained in the Intracellular calcium modeling section.

#### SK channels modeling

Small conductance calcium-regulated potassium channels are voltage-independent and regulated solely by calcium concentration in a microdomain surrounding the channel [46]. Therefore, they require a different calcium mapping compared to BK channels (see Intracellular calcium modeling section). Steady-state activation depends on intracellular calcium through a classical Hill function:

$$\begin{array}{c} m_{\text{KCNL},\infty }(Ca)=\frac{Ca}{K_{Ca}+Ca}\;, \end{array}$$ (24)

where *K*_*Ca*_ is the dissociation constant, equal to 0.33*μ*M [66, 67], and the Hill coefficient is assumed equal to 1 [68].

The total current flowing through SK channels is given by:

$$\begin{array}{c} I_{\text{KCNL}}={\bar{g}}_{\text{KCNL}}\cdot m_{\text{KCNL}}\cdot \left(V-V_{K}\right)\;. \end{array}$$ (25)

#### Intracellular calcium modeling

To take into account the effect of calcium concentration on channels gating, the intracellular calcium dynamics is modeled following two different approaches, each of them specific to properly describe calcium regulatory action on BK channels or SK channels.

For BK channels, the calcium behavior is described at the nanoscale, because the behaviour of each channel is tuned by one or more CaV, localized within a nanometric distance from the BK channel [50]. We model the intracellular calcium concentration ${\left[{\text{Ca}}^{2+}\right]}_{i}^{n}$ (where *n* denotes the nanoscale) inside a spherical nanodomain (*r* ∼ 10 nm) centered on the CaV, using reaction-diffusion equations in the steady-state excess buffer approximation (EBA) [51, 52, 69]. In these conditions, the ${\left[{\text{Ca}}^{2+}\right]}_{i}^{n}$ concentration in the proximity of an open *(o)* CaV is given by

$$\begin{array}{c} {\left[{\text{Ca}}^{2+}\right]}_{i,o}^{n}=\frac{i_{Ca}}{8\pi rD_{Ca}F}\exp[\frac{-r}{\sqrt{\frac{D_{Ca}}{k_{B}^{+}{\left[B\right]}_{\text{tot}}}}}], \end{array}$$ (26)

where *D*_*Ca*_ = 250*μ*m^2^ s^-1^ is the calcium diffusion coefficient, *r* = 13 nm the radius of the nanodomain, *F* = 96485 C mol^-1^ the Faraday constant, $k_{B}^{+}=500\;\mu {\text{M}}^{-1}{\text{s}}^{-1}$ is the intracellular buffer rate constant, [*B*]_tot_ = 30 *μ*M is the total intracellular buffer concentration, assumed constant over time as required by the EBA. The values of the parameters mentioned above are taken from [51], due to the absence of specific data for *C. elegans*. *i*_*Ca*_ is the calcium current through a single open voltage-gated calcium channel:

$$\begin{array}{c} i_{Ca}=g_{\text{sc}}\cdot \left(V-V_{Ca}\right)\;, \end{array}$$ (27)

where *g*_sc_ is the single channel conductance, assumed equal to 40 pS for both L-type and P/Q-type calcium channels [57], and *V*_*Ca*_ = 60 mV is the Nernst potential for calcium. When the calcium channel is closed (*c*) we assume a calcium concentration ${\left[Ca^{2+}\right]}_{i,c}^{n}$ equal to 0.05 *μ*M [21]. We remark that ${\left[{\text{Ca}}^{2+}\right]}_{i,o}^{n}$ and ${\left[{\text{Ca}}^{2+}\right]}_{i,c}^{n}$ define through Eqs 10 and 11 the parameters $k_{o}^{+/-}$ and $k_{c}^{-}$ which affect steady-state and time constant activation of BK within the complex.

In SK channels, activation and inactivation are modulated by intracellular calcium transients in a microdomain surrounding the channel [50]. Since an exhaustive experimental characterization of cytosolic calcium transport and diffusion is still missing for *C. elegans* neurons, we model the calcium dynamics at the microscale based on a simplified mass balance equation [55, 68]. We take into account calcium inflow through voltage-sensitive channels (*I*_*Ca*_) and outflow due to membrane exchangers removal, such as SERCA, Na^+^-Ca^2+^ exchangers, and plasma membrane Ca^2+^-ATPases [70]. In addition, we set an equilibrium concentration equal to a physiological baseline in zero current conditions, discarding source terms in case of current inversion to avoid concentrations lower than the baseline. This switching dynamics models compensatory cellular mechanisms avoiding too low intracellular calcium:

$$\begin{array}{ccc} \frac{d{\left[{\text{Ca}}^{2+}\right]}_{i}^{m}}{dt} & = & \left[1-H(V-V_{Ca})][-f\alpha I_{Ca}-(\frac{{\left[{\text{Ca}}^{2+}\right]}_{i}^{m}-{\left[{\text{Ca}}^{2+}\right]}_{\text{eq}}^{m}}{\tau_{Ca}})\right]\\ & & +H\left(V-V_{Ca}\right)(-\frac{{\left[{\text{Ca}}^{2+}\right]}_{i}^{m}-{\left[{\text{Ca}}^{2+}\right]}_{\text{eq}}^{m}}{\tau_{Ca}})\;, \end{array}$$ (28)

$$\begin{array}{ccc} \alpha & = & \frac{1}{2V_{\text{cell}}F}\;, \end{array}$$ (29)

$$\begin{array}{ccc} I_{Ca} & = & I_{\text{EGL19}}+I_{\text{UNC2}}+I_{\text{CCA1}}\;. \end{array}$$ (30)

${\left[{\text{Ca}}^{2+}\right]}_{i}^{m}$ denotes the microscale intracellular calcium concentration, *V*_cell_ represents total cellular volume, including soma axon and dendrites (values for AWC^ON^ and RMD are taken from the Neuromorpho database, http://neuromorpho.org/, see S1 Table), *α* is a conversion factor, *F* is the Faraday constant, *f* = 0.001 is the free intracellular calcium fraction, *τ*_*Ca*_ = 33 ms represents the calcium removal time constant, *H*(*V*) denotes the standard Heaviside function and *V*_*Ca*_ = 60 mV is the reversal potential for calcium. ${\left[{\text{Ca}}^{2+}\right]}_{\text{eq}}^{m}={\left[{\text{Ca}}^{2+}\right]}_{i,c}^{n}$ is the baseline calcium concentration, assumed equal to 0.05 *μ*M [21].

### Numerical implementation and data analysis

We implemented the whole model in XPPAUT [71], integrating the dynamical system with a “stiff” solver (based on Rosenbrock discretization schemes), known to be the preferred option when dealing with coupled dynamics characterized by different timescales [72]. We set restrictive values of numerical tolerances (∼ 1e-8) to ensure accurate results. Further restrictions on such setting did not show differences in numerical results indicating that we achieved a suitable accuracy. We used AUTO within XPPAUT for the computation of bifurcation diagrams. AUTO is a well-assessed tool for solving continuation problems to trace systems solution and bifurcations upon parameters variations.

Experimental data and simulations results were analyzed in MATLAB. Parameters identification for each channel, except for BK (see below) and SK (not requiring any fitting), was performed separately for each function, i.e. for steady-state activation, inactivation and time constant, leading to the joint fitting of three parameters on average (five at maximum). We used a least-squares nonlinear fitting (“lsqcurvefit” in MATLAB) by adopting a Trust-Region-Reflective algorithm, a choice suitable for bound constraints. Despite the possible identification of local minimizers instead of a global one, fitted parameters were both reasonable in their values and faithfully reproduced experimental data. For the BK channels model (SLO-1 [41] and SLO-2 [42]), the parameters identification was performed *via* a global optimization with a hybrid genetic algorithm [51], by fitting for each channel the steady-state activation and time constant simultaneously. Such algorithm is designed to identify global minimizers through a stochastic sampling of parameters and by their successive refinement over successive iterations (generations). We used the MATLAB function “ga” of the Optimization Toolbox, by setting the number of generations at 100 multiplied by the number of parameters to be estimated (eight parameters, i.e. $w_{0}^{+}$, $w_{0}^{-}$, *w*_*yx*_, *w*_*xy*_, *n*_*yx*_, *n*_*xy*_, *K*_*yx*_, and *K*_*xy*_). The identified parameters were further refined through the hybrid function “fmincon” with the “sqp” algorithm, based on sequential quadratic programming. The hybrid function, which is more efficient for local minimization, starts from the final point calculated by “ga”. In summary, the genetic algorithm identifies a global minimizer which is refined locally by the hybrid function. We performed 150 random trials, obtaining different sets of parameters well reproducing experimental data, among which we selected the best (by means of least squared errors) both for SLO-1 and SLO-2. This approach was shown to give accurate results in case of similar fitting of BK kinetics [51], with a composite objective function to be minimized over different datasets. Specifically, the algorithm minimizes the following objective function:

$$\begin{array}{ccc} min_{\text{slo-1/2},\;j}^{\phi } & = & \sum_{j}\sum_{i}{\left[m_{\text{BKs},\infty }^{\;j}\left(V_{i}\right)-{\bar{m}}_{\text{BKs},\infty }^{\;j}\left(V_{i}\right)\right]}^{2}\\ & & +{\left[\tau_{m_{\text{BKs}}}^{\;j}\left(V_{i}\right)-{\bar{\tau }}_{m_{\text{BKs}}}^{\;j}\left(V_{i}\right)\right]}^{2}\;, \end{array}$$ (31)

where *ϕ* identifies the parameter set, $m_{\text{BKs},\infty }^{\;j}$ and $\tau_{m_{\text{BKs}}}^{\;j}$ are the experimental points for j-th calcium concentration (*Ca* = 1, 10, 100, 1000 *μ*M for SLO1 [41], and *Ca* = 10, 60, 300 *μ*M for SLO2 [42]), and ${\bar{m}}_{\text{BKs},\infty }^{\;j}$ and ${\bar{\tau }}_{m_{\text{BKs}}}^{\;j}$ are the corresponding simulated equilibrium activation variable and time constant. In the case of SLO-1 channels, we sightly modified the objective function due to the lack of experimental data on activation time constants at *Ca* = 1*μ*M.

Analysis of voltage clamp simulation results is performed by calculating steady-state current-voltage (I-V) curves. Such curves are computed by averaging over the last 5 ms the current elicited by a single voltage step.

## Results and discussion

In this section, we initially present results for specific ionic currents and then we apply the whole-cell neuron model to the two neurons AWC^ON^ and RMD. Single ionic currents are discussed in details and their physiological role within the entire cell dynamics is analyzed based on wild type and *in silico* knockout simulations. We refer to current activation/inactivation upon stimulation as the activation/inactivation of the corresponding channels, as used in literature [15].

In our description, we report the genes encoding for membrane channels and the corresponding channel proteins in italic and capital roman letters, respectively. Ionic currents are named without the dash between the gene name and the number (e.g. *egl-19* gene encodes for EGL-19 channels that carry EGL19 current).

**Fig 1:**
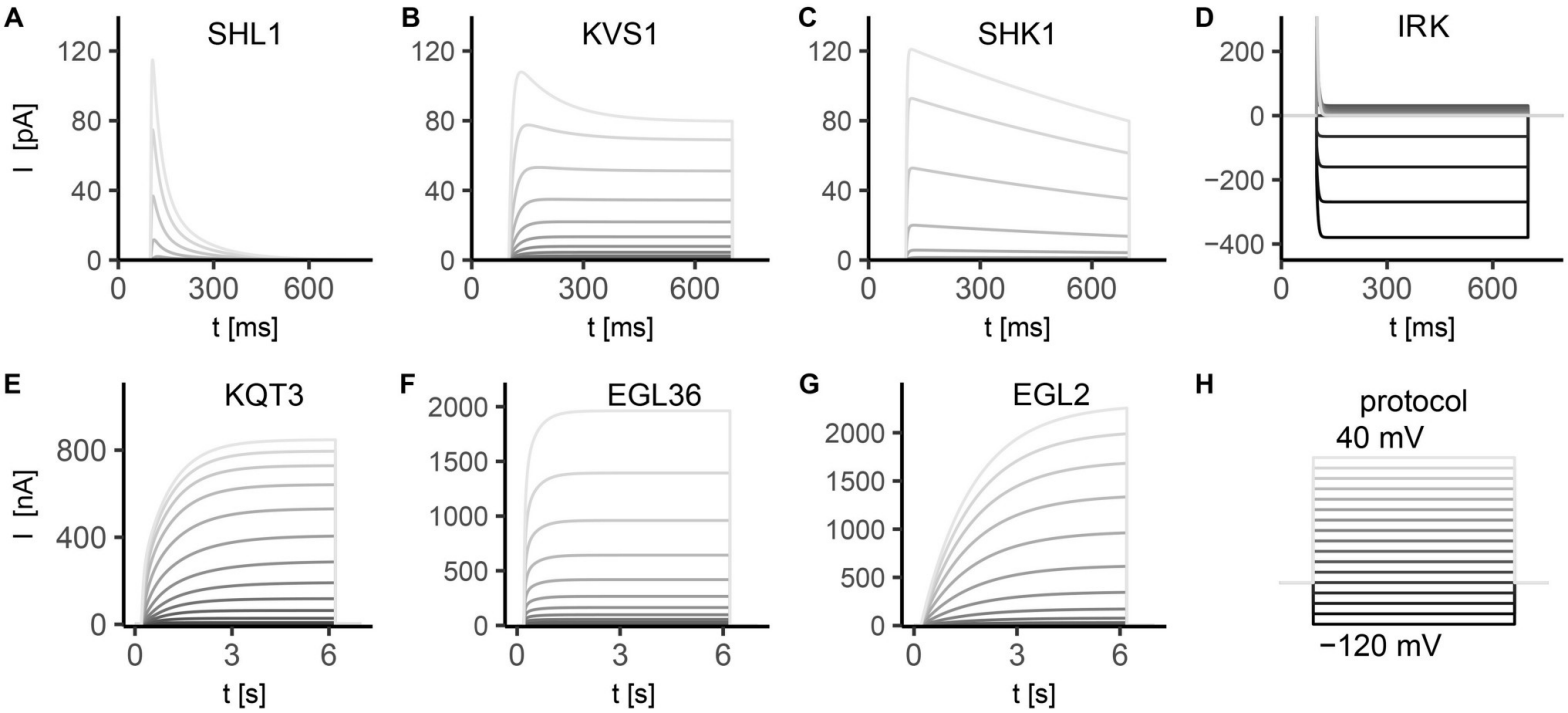
Simulated currents of the voltage-gated potassium channels SHL-1, KVS-1, SHK-1, IRK-1/3, KQT-3, EGL-36 and EGL-2. Potassium currents present two different timescales for activation: some of them have fast activation (panels A-D, $\tau_{m_{x}}<15\;\text{ms}$), others have slow activation (panels E-G, ∼100 ms $<\tau_{m_{x}}<2\;\text{s}$). Stimulation protocol (sketched in panel H) consists in 10 mV voltage steps, from -120 mV to 40 mV. Each step lasts 600 ms for fast activating currents (panels A-D) and 6 s for slow activating ones (panels E-G). The holding potential is assumed equal to potassium reversal potential, i.e. *V*_*h*_ = −80 mV. The currents are expressed in pA (panels A-D) or nA (panels E-G), depending on the experimental data used as reference. The simulated currents in panels A-G are given by Eqs A5, A9, A14, A30, A21, A27, and A24 in S1 File, respectively, in which the conductances are assumed as follows: ${\bar{g}}_{\text{SHL}1}=1.8\;\text{nS}$, ${\bar{g}}_{\text{KVS}1}=3\;\text{nS}$, ${\bar{g}}_{\text{SHK}1}=1.1\;\text{nS}$, ${\bar{g}}_{\text{IRK}}=10\;\text{nS}$, ${\bar{g}}_{\text{KQT}3}=36.5\;\text{nS}$, ${\bar{g}}_{\text{EGL}36}=50\;\text{nS}$, and ${\bar{g}}_{\text{EGL}2}=20\;\text{nS}$. **A) SHL1 current.** Experimental data from [25]. Activation time constant is $\tau_{m_{\text{SHL}1}}∼18\;\text{ms}$ at *V* = −10 mV (Eq A2 in S1 File, S1 Fig). Current inactivation is described by the sum of two exponential functions, with time constants $\tau_{h_{\text{SHL}1}}^{f}∼40\;\text{ms}$ and $\tau_{h_{\text{SHL}1}}^{s}∼200\;\text{ms}$ at *V* > −10 mV for fast and slow components (Eq A4 in S1 File, S1 Fig). **B) KVS1 current.** Experimental data from [31]. KVS1 current activates significantly at positive potentials, with a time constant $\tau_{m_{\text{KVS}1}}∼20\;\text{ms}$ at 0 mV (Eq A8 in S1 File, S1 Fig). The current inactivates exponentially with a time constant that is a decreasing function of voltage ($\tau_{h_{\text{KVS}1}}∼130\;\text{ms}$ at 0 mV, S1 Fig). **C) SHK1 current.** Experimental data for steady-state activation and inactivation variables from [25], and from [29] for time constants. SHK1 current shows fast activation for depolarizing potentials ($\tau_{m_{\text{SHK}1}}∼5\;\text{ms}$ at 0 mV, Eq A11 in S1 File), followed by exponential inactivation with a voltage independent time constant $\tau_{h_{\text{SHK}1}}=1400\;\text{ms}$ (Eqs A12 and A13 in S1 File, S2 Fig). **D) IRK current.** Experimental data from [33–35]. The activation kinetics is described by a Boltzmann function (Eq A28 in S1 File) that is almost null at positive potentials and saturates at very negative potentials (*V* < −100 mV) (S2 Fig). The activation time constant voltage dependence is described by an asymmetric bell-shaped curve (Eq A29 in S1 File) with a peak of 8 ms at ∼-40 mV, and decreases to values below 4 ms for positive voltages (S2 Fig). **E) KQT3 current.** Experimental data from [26]. KQT3 current is characterized by the sum of two exponential functions with fast ($\tau_{m_{\text{KQT}3}}^{f}∼30\;\text{ms}$ at *V* ∼ −20 mV, Eq A16 in S1 File) and slow ($\tau_{m_{\text{KQT}3}}^{s}∼140\;\text{ms}$ at *V* ∼ −20 mV, Eq A17 in S1 File) activation components that account respectively for 30% and 70% of the total current (see S1 Fig). Inactivation is described by Eq A18 in S1 File [73]. **F) EGL36 current**. Experimental data from [30]. The current activates at high voltages *V* > 10 mV (S2 Fig) with three almost equally-weighted components at fast (13 ms), medium (63 ms) and slow (355 ms) timescales. Steady-state activation variable (Eq A25 in S1 File, S2 Fig) is shifted towards lower potentials (*V*_0.5_ = 6.9 mV) compared to SHL1, SHK1 and KVS1. **G) EGL2 current**. Experimental data from [28]. EGL2 current is slow activating ($\tau_{m_{\text{EGL}2}}∼1\;\text{s}$) and non-inactivating. The activation function is described by a Boltzmann function with slow rise (*k*_*a*_ = 14.9 mV) between -40 mV and 40 mV (Eq A22 in S1 File, S2 Fig). Activation time constant is weakly dependent on voltage, becoming almost constant for the considered voltages (Eq A23 in S1 File, S2 Fig). **H) Simulation protocol.** 10 mV voltage steps, range -120 mV to 40 mV, step duration 600 ms or 6 s.

**Fig 2:**
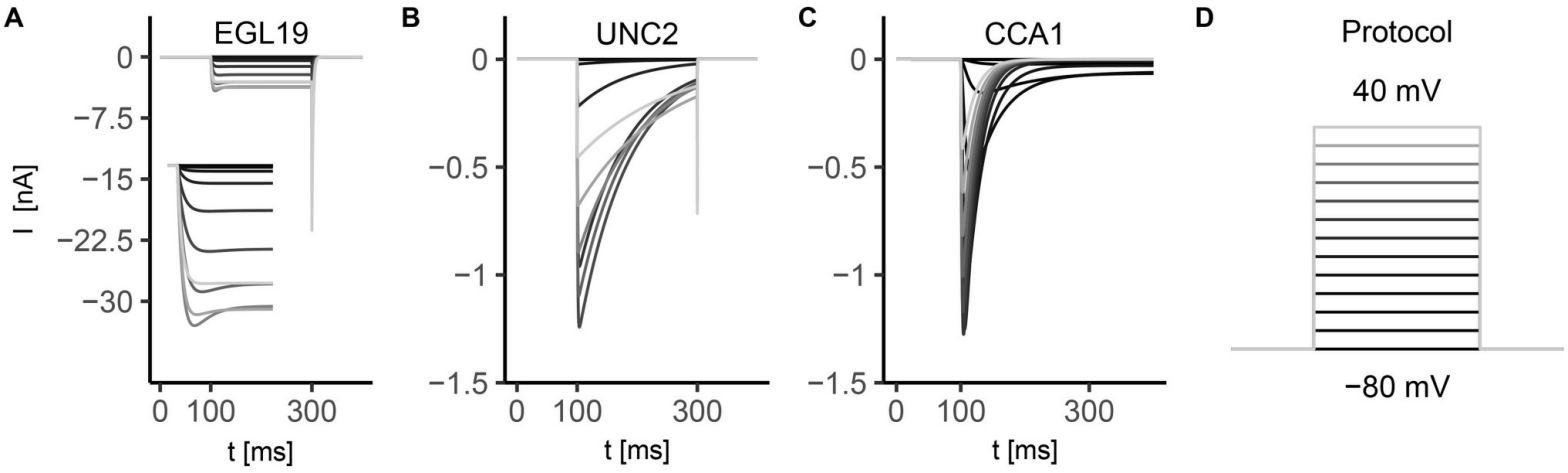
Simulated currents of voltage-gated calcium channels EGL1-9, UNC-2, and CCA-1. Calcium currents can be classified according to their activation voltage level: EGL19 and UNC2 currents activate at high membrane potential (*V* > ∼ − 40 mV), while CCA1 currents start to activate at low voltages (*V* ∼ −70 mV) [15, 20, 36, 37, 75]. Stimulation protocol (sketched in panel D) consists in 10 mV voltage steps ranging from -80 mV to 40 mV. Each step lasts 200 ms, and is applied from a holding potential *V*_*h*_ = −80 mV. The reversal potential for calcium is *V*_*Ca*_ = 60 mV. EGL19, UNC2, and CCA1 currents are given by Eqs B5, B10, and B14 in S1 File, respectively, in which ${\bar{g}}_{\text{EGL}19}=200\;\text{nS}$, ${\bar{g}}_{\text{UNC}2}=23\;\text{nS}$, and ${\bar{g}}_{\text{CCA}1}=25\;\text{nS}$ (conductance values are chosen to match the currents of reference experimental data). The currents are expressed in nA, depending on the experimental data used as reference. **A) EGL19 current**. Experimental data for steady-state activation and inactivation variables from [15] and for activation and inactivation time constants from [36]. EGL19 current activates rapidly ($\tau_{m_{\text{EGL}19}}∼6\;\text{ms}$ at 0 mV) at high voltage (*V* > −30 mV, see S3 Fig). The voltage dependence of the activation time constant is described by the sum of two Gaussian functions with shifted centers (Eq B2 in S1 File and S3 Fig), as in [76]. The steady-state inactivation function has a U-shape, with a minimum of about 0.5 at 0 mV (Eq B3 in S1 File and S3 Fig). The inactivation time constant voltage dependence is described by the sum of two sigmoids (Eq B4 in S1 File and S3 Fig), as in [76]. **B) UNC2 current**. Experimental data for steady-state activation and inactivation curves from [37], and for activation and inactivation time constants from [38] and [39], respectively. UNC2 current starts to activate at voltages slightly lower than in the case of EGL19 (*V*_0.5_ ∼ −10 mV and ∼6 mV for UNC2 and EGL19, respectively), with a steady-state activation function steeper than EGL19 (*k*_*a*_ ∼ 4 mV and ∼8 mV for UNC2 and EGL19, respectively) (Eq B6 in S1 File and S3 Fig). The current shows fast activation with time constant voltage dependence described by a bell-shaped curve (Eq B7 in S1 File and S3 Fig) with a maximum value $\tau_{m_{\text{UNC}2}}=0.8\;\text{ms}$ at around -10 mV. Inactivation time constant is described by two sigmoids with ∼90 ms $<\tau_{h_{\text{UNC}2}}<∼150\;\text{ms}$ (Eq B9 in S1 File, and S3 Fig). **C) CCA1 current.** Experimental data from [40]. CCA1 current exhibits a fast activation with a time constant $\tau_{m_{\text{CCA}1}}<10\;\text{ms}$ for *V* > −40 mV, followed by an inactivation with a time constant $\tau_{h_{\text{CCA}1}}∼20\;\text{ms}$ for *V* > −30 mV. The current activates at more negative potentials than UNC2 and EGL19 currents (*V*_0.5_ ∼ −60 mV) (Eq B11 in S1 File and S3 Fig), and the steady-state activation and inactivation curves overlap between -70 mV and -30 mV giving rise to a sustained inward current, named window current (S3 Fig). **D) Stimulation protocol.** 10 mV voltage steps, range -80 mV to 40 mV, step duration 200 ms.

**Fig 3:**
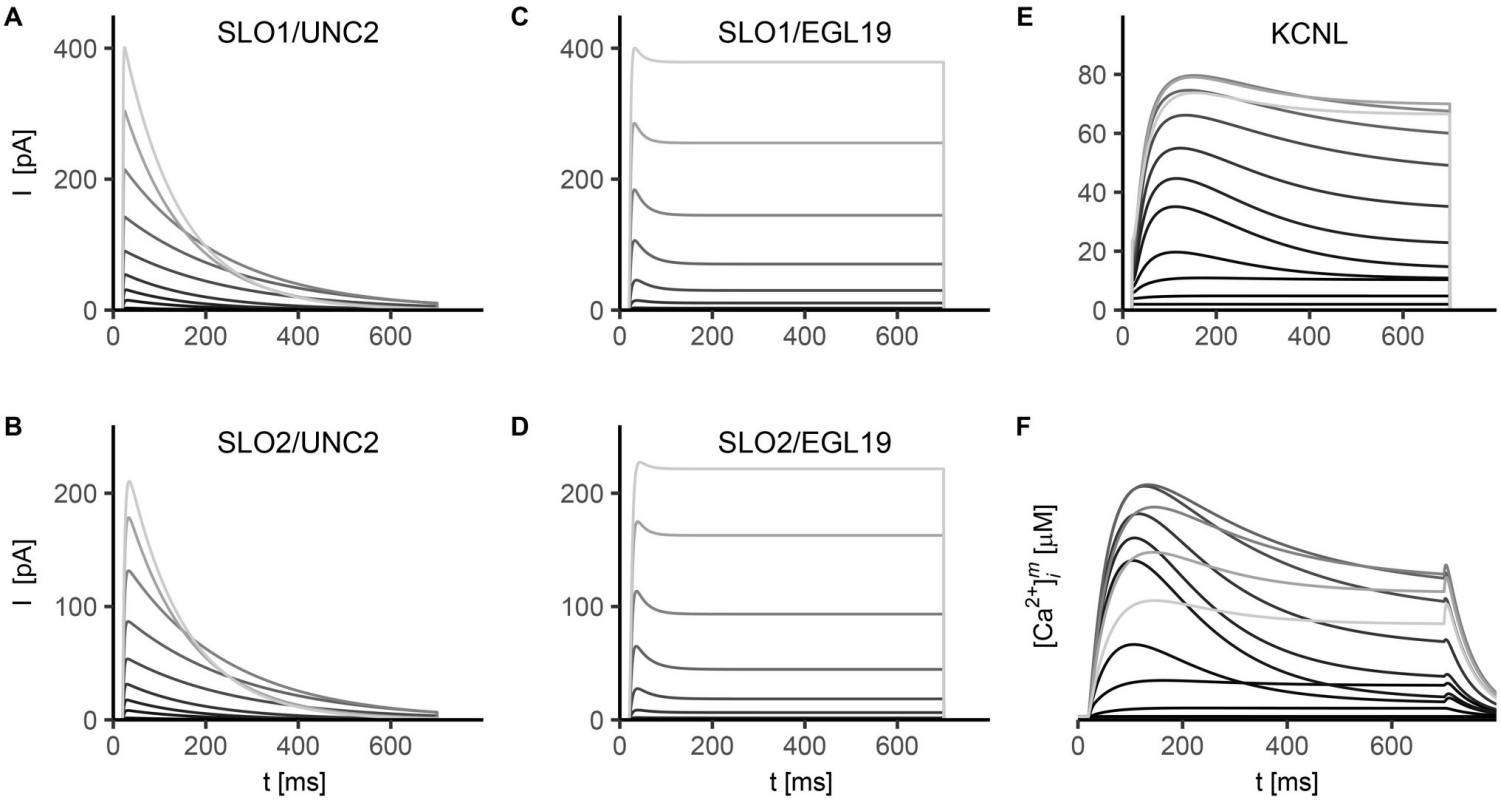
Simulated currents of calcium-regulated potassium channels SLO-1, SLO-2 and KCNL. Stimulation protocol consists in 10 mV voltage steps ranging from -80 mV to -40 mV. Each step lasts 600 ms, and is applied from a holding potential *V*_*h*_ = −80 mV. For both SLO1 and SLO2 we set the conductance to 15 nS (Eq 1). For KCNL current the conductance is ${\bar{g}}_{\text{KCNL}}=1.5\;\text{nS}$. The modified set of parameters, i.e. fine-tuned to match neuron data (see S1 Table), has been used for CaV channels dynamics. **A-B) SLO1 (A) and SLO2 (B) coupled with UNC-2 Ca^2+^ channels**. SLO1 and SLO2 simulated currents show fast activation ($\tau_{m_{\text{SLO}1}}∼8\;\text{ms}$, and $\tau_{m_{\text{SLO}2}}∼5\;\text{ms}$, Eq 22). Current activation is followed by inactivation shaped by the inactivation variable of the coupled UNC2 current (Eq 23). **C-D) SLO1 (C) and SLO2 (D) coupled with EGL-19 Ca^2+^ channels**. Analogously to panels A-B the currents show fast activation, while partial inactivation is strictly related to the limited inactivation of EGL19 calcium current (Eq 23, Fig 2A and S3 Fig). **E) KCNL current**. Experimental data from [67, 68]. KCNL channels exhibit slow activation ($\tau_{m_{\text{KCNL}}}=6.3\;\text{ms}$) and inactivation. The steady-state activation function depends only upon intracellular calcium concentration (Eq C6 in S1 File and S5 Fig). **F) Simulated intracellular calcium concentration**. ${\left[{\text{Ca}}^{2+}\right]}_{i}^{m}$ during voltage clamp simulation is calculated by solving Eq 28, where *I*_*Ca*_ is given by *I*_EGL19_ + *I*_UNC2_ + *I*_CCA1_ with ${\bar{g}}_{\text{EGL}19}={\bar{g}}_{\text{UNC}2}={\bar{g}}_{\text{CCA}1}=1\;\text{nS}$.

All the simulated ionic currents are shown in Figs 1, 2 and 3, according to their ion specificity and gating mechanism (KV, CaV, KCa). Fitted parameters, as identified by least-squares nonlinear fitting, are reported in S1 Table, and we rely on the value of these parameters in the following description and discussion of results for single channels. Steady-state activation/inactivation curves and time constant functions computed with the fitted parameters are shown in S1, S2, S3, S4 and S5 Figs. The conductance for each channel was set to reproduce peak and steady-state current values as measured in experiments (see Figure captions).

For some currents, we based our modelling on channels expressed in *C. elegans* muscle cells or on homologous channels expressed in other organisms. In these cases, we *a posteriori* calibrated a subset of the fitted parameters for the whole-cell model in order to match data on *C. elegans* neurons, i.e. voltage clamp data—analyzing whole membrane current—and current clamp data [13, 59]. Specifically, we carefully shifted half-activation and inactivation potentials and time constants of some of the modeled currents (SHL1, KVS1, KQT3, EGL2, EGL19, UNC2, CCA1) to correctly reproduce timescales and voltage sensitivity as observed in neurons (see S1 Table). Calibrated parameters are reported in parentheses in S1 Table. The conductances set was tuned to match whole-cell data (see Table 2).

**Table 2:** Ionic conductances for AWC^ON^ and RMD neurons. We use *V*_*K*_ = −80 mV, *V*_*Ca*_ = 60 mV and *V*_*Na*_ = 30 mV as the reversal potentials for potassium, calcium and sodium currents, respectively. In AWC^ON^ the reversal potential for leakage current (Eq C10 in S1 File) is *V*_*L*_ = −90 mV; in RMD it is *V*_*L*_ = −80 mV.

*Current*	*Conductance* (${\bar{g}}_{x}$) [nS]	
	AWC^ON^	RMD
SHL1	2.9	2.5
SHK1	0.1	1.1
KVS1	0.8	-
EGL2	0.85	-
EGL36	-	1.3
KQT3	0.55	-
EGL19	1.55	0.99
UNC2	1	0.9
CCA1	0.7	3.10
SLO1/EGL19	0.11	0.30
SLO1/UNC2	0.11	0.30
SLO2/EGL19	0.10	0.3
SLO2/UNC2	0.10	0.3
KCNL	0.06	0.06
NCA	0.06	0.05
IRK	0.25	0.20
LEAK	0.27	0.40

In Fig 1, all the currents for voltage-gated potassium channels are reported. Most of the voltage-gated potassium channels are conserved among species [74] due to their fundamental role in regulating neurons and muscles electrical activity by governing excitability of tissues, setting the resting potential and shaping the action potential. The majority of channels modeled here is activated by depolarization and the heterogeneity of their kinetics reflects the wide range of their biological role. In Fig 1, panels A-D, we report ionic currents of K^+^ channels with fast dynamics, on the scale of few milliseconds for activation and few hundreds of milliseconds for inactivation. Their role is pivotal in controlling the rise time of membrane depolarization [10, 17], moreover SHK-1 and SHL-1 channels are the main responsible of the outward current in *C. elegans* muscle cells [18]. In SHL1, both the fast and slow inactivation time constants are decreasing with membrane depolarization, and steady-state activation and inactivation curves are strongly voltage-dependent [25] (Fig 1A and S1 Fig). In response to voltage steps, KVS1 current activates at positive potentials and show almost no inactivation at negative potentials (Fig 1B and S1 Fig). SHK1 current rapidly activates and slowly inactivates in response to voltage steps [25] (Fig 1C and S2 Fig). Steady-state activation and inactivation curves are shifted to more positive potentials compared to SHL1 current, indicating a very low contribution of SHK1 at negative potentials to the overall K^+^ current. IRK channels (Fig 1D and S2 Fig) give rise to sustained fast inward rectifying current at potentials near or below the reversal potential for potassium. Panels E-G of Fig 1 report the ionic currents for K^+^ channels with very slow activation timescales, requiring up to 1 second to reach the steady-state. KQT3 shows almost absent inactivation while EGL2 and EGL36 are characterized only by activation dynamics (Eqs A22 and A25 in S1 File respectively), being channels contributing to cell repolarization. EGL36 is also characterized by a complex interplay of three timescales in the activation dynamics (see caption of Fig 1), with the three time constants almost insensitive to voltage [30].

In Fig 2, we report the currents for voltage-gated calcium channels. Calcium ions are the primary mediators of depolarization-induced calcium entry and are crucial for cell signalling [77]. Opening of voltage-gated calcium channels greatly increases the concentration of intracellular calcium (from nanomolar to micromolar range) and initiates a wide-range of calcium-dependent processes like neurotransmitter release, gene transcription, activation of enzymes. We modeled three currents, EGL19, UNC2 and CCA1, representative of the three main classes of CaV in *C. elegans*: L-type, P/Q-type and T-type respectively, that are expressed in the considered neurons (see Table 1). EGL-19 channels, the only L-type channels in *C. elegans*, activate at high voltage and partially inactivate at membrane potential values between -40 mV and 40 mV (Fig 2A). Due to their voltage-dependence and their limited inactivation, EGL19 current may significantly contribute to the plateau phase of the action potential. UNC2 current (Fig 2B) activates at intermediate voltage values with respect to EGL19 and CCA1, and is characterized by fast activation and slow inactivation. In Fig 2C, we report CCA1 current which is characterized by fast activation and inactivation kinetics with a strong voltage dependence of inactivation. CCA-1 channels (also known as low-voltage-activated channels) activate in response to small depolarizations compared to P/Q- and L-type (S3 Fig, Eqs B11 and B12 in S1 File) [75].

In Fig 3 we report the simulated currents for the potassium channels regulated by intracellular calcium concentration (KCa). KCa are largely expressed in the nervous system and they are involved in regulating excitability of soma, synaptic transmission and plasticity [46, 47]. Apart from the main differences between BK and SK channels (BK are co-regulated by calcium concentration and voltage, while SK are voltage independent), already included in our model (see Methods), they also differ in their sensitivity to calcium changes. SK channels are usually active at physiological calcium concentrations and very sensitive to small changes while BK channels are normally activated at very high calcium concentrations, reached only in the proximity of voltage-gated calcium channels [50].

Concerning BK, as a first step, we fine-tuned voltage and calcium sensitivity based on isolated SLO channels data [41, 42]. In S1 Table we report the best fitting parameters obtained after multiple runs of the genetic algorithm. The resulting activation variable curves, time constant curves and simulated currents are shown in S4 Fig. Most of the fitted parameters are consistent with reported values of BK channels kinetics fine-tuned on human HEK cells lines [51]. This could indicate that voltage sensitivity, in opening and closing transitions, is similar in BK channels and in homologous channels expressed in different organisms. Notably, opening transition rate at 0 mV for SLO-1 is 3.15 ms compared to the reported value of 3.32 ms in HEK cells. The computed values of Hill coefficients for SLO also suggest a conserved positive cooperative binding in channel opening and a more pronounced negative cooperative binding in channel closing. In addition, in SLO channels opening, we obtain a half-activation value (*K*_*xy*_), in response to calcium, enhanced with respect to HEK. In the case of SLO-2 closing, the half-activation value (*K*_*yx*_) in response to calcium is several orders of magnitude higher than the same value calculated for SLO-1 and reported for HEK. This result can be related to a significantly different affinity for calcium in opening and closing transitions. However, we cannot exclude that such a difference could also depend on data used to fine-tune the model, therefore other experimental sets would be required to validate our findings.

The parameters obtained from the isolated BK model are incorporated in the BK-CaV complex description, and in Fig 3, panels A-D, we report the simulated SLO1 and SLO2 currents. BK channels in *C. elegans* are encoded by *slo-1* and *slo-2* genes, which show an overlapping expression pattern in muscles and neurons, where the corresponding channels colocalize with EGL-19 and UNC-2 channels [53, 54]. This co-expression is related to the activation mechanism for BK. They are indeed almost closed at rest, and the calcium concentration required for activation is reached only in the proximity of a voltage-activated calcium channel [50]. Therefore, BK channels are usually strictly coupled with one or more CaV, with the two types of channels colocalized within a nanodomain (of radius *r* ∼ 10 nm). When the coupled CaV activates, the intracellular calcium rises and causes a shift towards more negative potentials of the activation curve of the BK channel, thereby allowing its activation in physiological conditions. We study the 1:1 coupling between SLO-1/SLO-2 and EGL-19/UNC-2. No experimental evidence is found for co-expression of BK channels with CCA-1. Concerning the additional regulation by intracellular chloride concentration for SLO-2 [14, 27, 42], it is still debated in literature if actually chloride regulates these channels, and in any case, no experimental data are available to eventually model the co-regulation. However, it is likely that such additional sensitivity does not contribute to single cell dynamics but it may have a significant role in regulating signal transmission, by working synergically with chloride channels such as the ones modulated by GABA. Despite *slo-1* and *slo-2* have an overlapping expression, similar functions and overall similarities in current behavior (in Fig 3, compare panels A-B, C-D), these channels also exhibit some differences in their kinetics of activation: SLO-2 channels show an increased half-activation potential at low calcium concentrations and present a lower time constant compared to SLO-1 (S4 Fig).

KCNL current, carried by small-conductance KCNL-1/3 channels, is shown in Fig 3E. This current is modeled as non-inactivating and with an activation variable dependent solely on calcium concentration (Eqs 24 and 25, S5 Fig). Specifically, KCNL steady-state activation function depends on intracellular calcium concentration ${\left[{\text{Ca}}^{2+}\right]}_{i}^{m}$, shown in Fig 3F (Eq C6 in S1 File, and S5 Fig). We highlight that intracellular calcium concentration reflects the voltage-dependent activation and inactivation of CaV which in turn affects the behavior of KCNL current. Therefore KCNL conductance is strictly linked to the intracellular calcium dynamics at the microscale. Notably, more sophisticated intracellular calcium models have been proposed in literature to simulate neuronal response to specific stimuli, like odorants [21–23]. However, based on the different stimulation protocol, experimental techniques (calcium imaging data), timescales analyzed and additional chemical feedbacks, such models can not be directly used to map intracellular calcium concentration shaping the electrical activity, as accomplished in the present work.

### Whole-cell neuron model

Here we present the models of two different types of neurons, a chemosensory neuron (AWC^ON^) and a motor neuron (RMD), providing a comprehensive description of their neuronal dynamics. We combined the ion currents described above based on gene expression profile (from Wormbase: https://www.wormbase.org), fine-tuning the conductance values (see Table 2) to reproduce the available experimental data, i.e. membrane potential and current wave forms. Our models are used to predict electrical behavior, when the corresponding experimental data are missing. In particular, we simulate wild type (WT) and *in silico* knockout neurons, dissecting each ion current and describing their specific role in the electrical dynamics of the two cells.

#### AWC^ON^ chemosensory neuron

**Fig 4:**
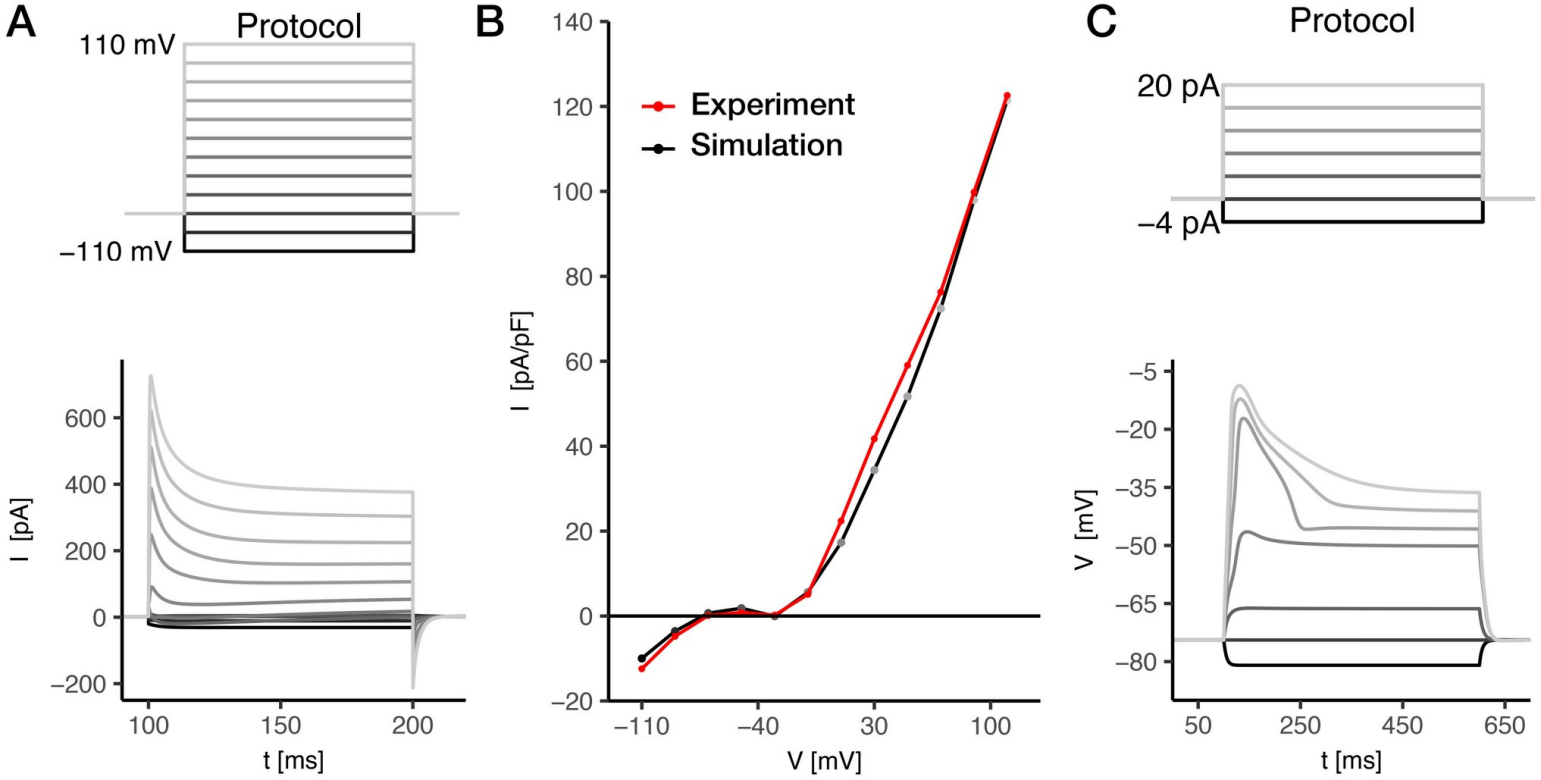
AWC^ON^ response to current and voltage stimuli. **A) Voltage clamp simulation of the AWC^ON^ neuron**. Experimental data from [59]. To test the model we apply to the *in silico* neuron the same voltage clamp stimulation protocol of experimental whole-cell recordings [59]. The protocol consists in voltage steps ranging between -110 mV and +110 mV with 20 mV increments. The holding potential is *V*_*h*_ = −70 mV, and the step duration is 100 ms. **B) AWC^ON^ steady-state I-V relation**. Comparison between experimental (red, data from [59]) and simulated (black) steady-state I-V curves. The simulated I-V curve is computed by averaging the currents in the last 5 ms of each voltage step, as in [59]. **C) Current clamp simulation of the AWC^ON^ neuron**. No published current clamp experimental data were found for AWC^ON^. The *in silico* neuron shows active behavior when the injected current is above 6 pA (S6 Fig). The voltage response is characterized by an upstroke with a duration of ∼30 ms for *I*_ext_ > 15 pA, followed by a plateau phase. The plateau height increases linearly with the stimulus amplitude (S6 Fig). A sensitivity analysis performed by varying the calcium removal rate *τ*_*Ca*_ (Eq 28 and S6 Fig) do not show significant changes in the results. Current clamp stimulation protocol consists in 6 current steps ranging from -4 to 20 pA, with a duration of 500 ms. The holding current is *I*_*h*_ = 0 pA. The cell capacitance is 3.1 pF [59], that corresponds to 1.3*μ*F/cm^2^ when scaled on the entire cell surface (*S*_*cell*_ = 238.16*μ*m^2^,from Neuromorpho.org). This value is in agreement with specific membrane capacitance reported for ASER [57], and with the one calculated for AIY by considering its total capacitance (∼0.7 pF [78]) and surface (65.89 *μ*m^2^, from Neuromorpho.org).

Voltage clamp simulations for AWC^ON^ are shown in Fig 4A, as obtained by relying on experimental data from [59]. In response to voltage pulses, from a holding potential of -70 mV, membrane currents are mainly inward with passive behavior at negative command potentials (≤ −60 mV), while they show growing outward peaks at increasing command potentials. At intermediate voltage pulse intensities (between −60 mV and −20 mV), the current decreases rapidly after the peak, reaching a local minimum before slowly increasing again to a stationary value (Fig 4A). Indeed, in the voltage range from −60 mV to −20 mV, a strong nonlinear response is clearly visible in the steady-state I-V relation (Fig 4B). This behavior is due to a complex interplay between outward potassium and inward calcium currents, further detailed below. Finally, for voltage > −20 mV, an almost linear I-V relation is recovered. Simulated membrane current is in agreement with the experimental one, and the model is used to predict membrane voltage dynamics upon current stimulation.

Current clamp simulations, performed by applying current stimuli from -2 pA up to 20 pA, reveal a threshold-like response in membrane voltage dynamics (Fig 4C), with a passive behavior at low amplitude stimuli and the appearance of regenerative responses at increasing current values, evoking a fast upstroke of the voltage followed by a slowly decreasing plateau. The overall response of AWC^ON^ to external currents resembles the real activation of this chemosensory neuron upon a chemical stimulus with a regenerative behavior [79].

**Fig 5:**
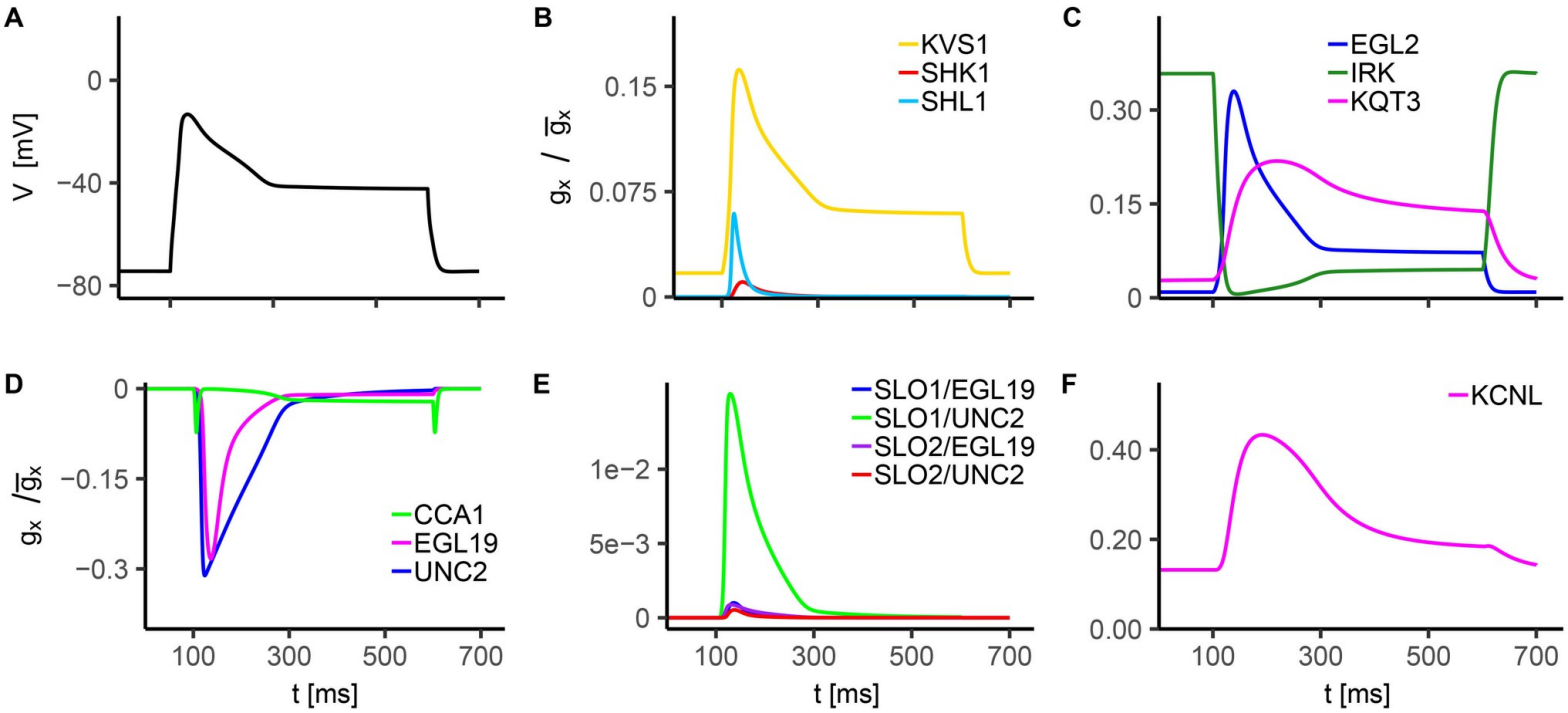
*In silico* analysis of AWC^ON^ voltage response to current injection. The stimulation protocol consists in one single 15 pA current step with a duration of 500 ms. The holding current is *I*_*h*_ = 0pA. **A) WT Voltage response to 15 pA current injection.** **B-F) Normalized conductance for modeled currents**. In these panels we report the time evolution of the normalized conductance of each modeled current for the WT current clamp simulation shown in panel A. The normalized conductance is defined as the product of the activation and inactivation variables of the considered ion current (e.g. for SHL1 $g_{x}/{\bar{g}}_{x}=m_{\text{SHL1}}^{3}\left(0.7\;h_{\text{SHL1}}^{\text{f}}+0.3\;h_{\text{SHL1}}^{\text{s}}\right)$). Panels B and C show $g_{x}/{\bar{g}}_{x}$ for voltage-gated K^+^ currents, panel D for voltage-gated Ca^2+^ currents, and panels E and F for calcium-dependent K^+^ currents. The $g_{x}/{\bar{g}}_{x}$ values for CCA1, UNC2 and EGL19 currents are multiplied by -1 to reproduce the sign of the associated currents.

In order to elucidate the ion channels role in the overall neuronal response upon current injection, we further analyze single current activation and inactivation dynamics (Fig 5A–5F) in wild type neuron.

A value of stimulating current of 15 pA has been selected, which ensures to reach the neuronal threshold, i.e. it is sufficient to evoke a large amplitude oscillation of membrane voltage. The initial fast depolarization is mainly due to inward calcium currents, with CCA1 and UNC2 lately supported by EGL19 (Fig 5D). The calcium currents also contribute to the plateau phase with particular regards to CCA1. Also, at the stimulus removal, the membrane voltage falls in a range suitable to achieve a partial reactivation of CCA-1 channels which induce a second peak in the L-type calcium current. This phenomenon suggests that CCA1 current is also involved in membrane repolarization. Outward potassium currents KVS1, SHL1, EGL2 and KQT3 counterbalance calcium currents. From their activation dynamics (Fig 5B and 5C), it can be argued that they affect membrane voltage upstroke, drive membrane repolarization and contribute to the plateau phase. Notably, the inward potassium current IRK shows significant activation levels before the application of the stimulus, in the early phase of depolarization and during the plateau, suggesting a role in tuning the resting potential, in shaping the action potential peak and in the regulation of the plateau phase. Among BK channels currents, the most intense one is SLO1/UNC2 (Fig 5E). However, the overall contribution of SLO currents is small. Indeed, only at very high calcium concentrations (at the nanoscale) SLO channels show significant steady-state activation levels (S4 Fig), while they are almost deactivated at lower concentrations. Therefore, in this case, calcium levels driving SLO channels may be too low to achieve a strong activation. Possibly, different condition of intracellular buffering or colocalization with more than one CaV could lead to robust activation of both SLO-1 and SLO-2 and this should be further explored in future investigations. On the other hand, a marginal role of SLO channels in shaping electrical activity was also found in motor neurons [14], although they have been shown to be important regulators of neurotransmitters release. KCNL seems to have a role similar to KQT3, affecting membrane repolarization after the peak and contributing to the plateau phase (Fig 5F).

**Fig 6:**
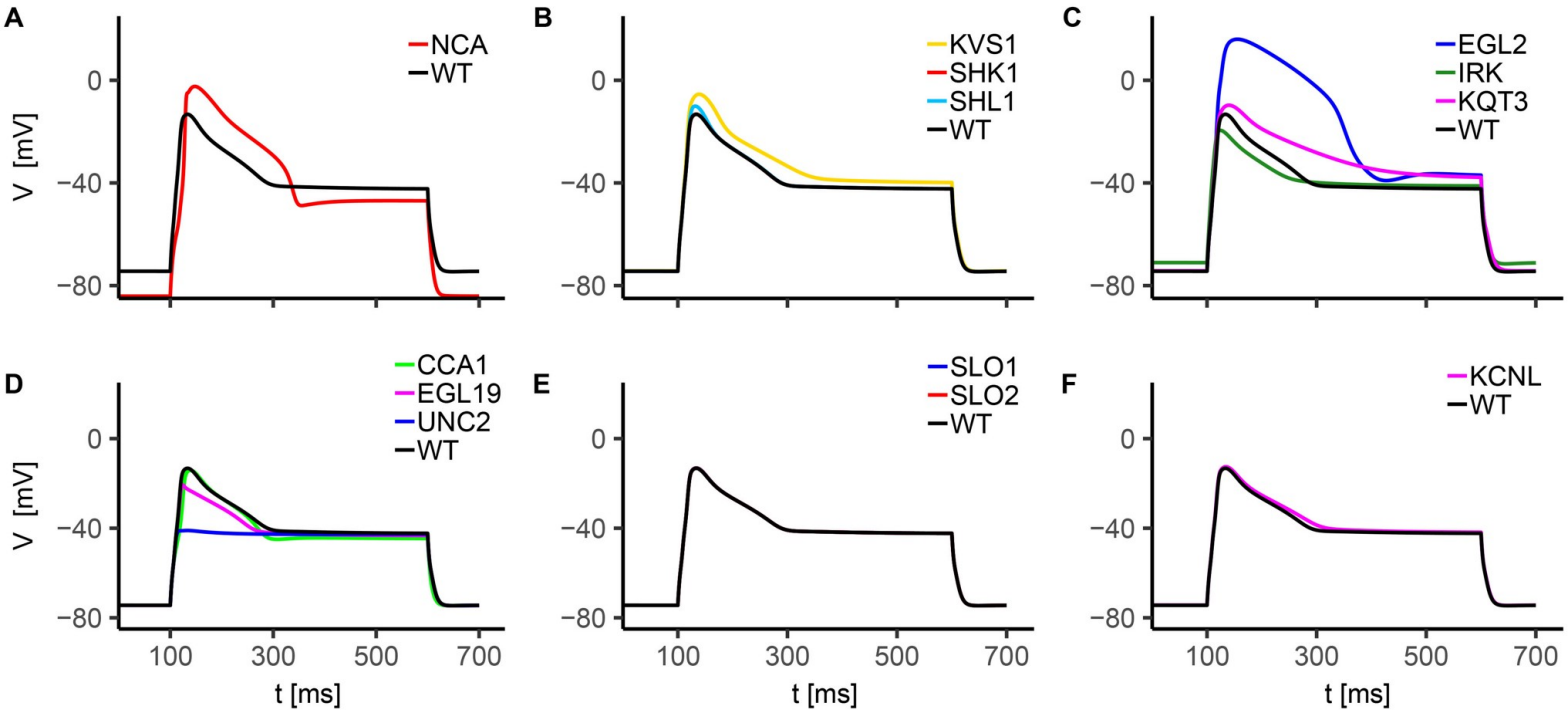
*In silico* AWC^ON^ knockouts voltage response to current injection. The stimulation protocol consists in a single 15 pA current step with a duration of 500 ms. The holding current is *I*_*h*_ = 0pA. Each knockout is obtained by suppressing the contribution of the selected current, leaving unchanged the other conductances. In panels A-F we report the response of NCA (A), voltage-gated potassium channels (B and C), voltage-gated calcium channels (D), and calcium-regulated potassium channels (E and F) knockouts.

The analysis of single currents in WT neuron is not exhaustive, because it does not reveal synergic activation and inactivation of ion channels induced by voltage and calcium feedback. To gain more insight, we model *in silico* knockout neurons, by selectively removing single ion currents from the WT model, thereby mimicking the effect of channel blocking. The membrane voltage dynamics corresponding to the different blockades are shown in Fig 6A–6F.

Notably, upon suppression of SHL1, KVS1, EGL2, and KQT3 currents, the neuron shows increased depolarization levels both in the peak and in the plateau regions, further confirming their role in balancing calcium current (see Fig 6B and 6C). In particular, EGL2 deletion strongly enhances membrane voltage peak and affects the repolarization phase before the plateau, indicating its major contribution compared to the other potassium currents. Calcium currents suppression show additional interesting features (Fig 6D). In particular, UNC2 deletion strongly suppresses threshold behavior and evokes a membrane potential waveform resembling a passive response, while the EGL19 knockout case presents a significant reduction in the upstroke of membrane potential (smaller compared to the UNC2 deletion case) and a slightly reduced plateau level due to an unbalancing of outward potassium. These observations support the prominent role of these two currents, especially of UNC2, both in the early and the late phases of membrane depolarization, as already inferred from single currents analysis (Fig 5D).

There are no electrophysiological data that directly measure the dynamics of AWC neurons in *unc-2* mutant worms, but UNC-2 channels have a pivotal role in controlling electrical activity as testified by defective locomotions, defective egg-laying [37], decreased number of spikes-per-train in body wall muscles and impaired neurotransmitter release [17] observed in *unc-2* mutant worms. Furthermore, these channels, together with EGL-19 channels, are key regulators in asymmetric AWC neurons differentiation [61, 80]. CCA1 current supports the upstroke phase, promoting the increase of membrane potential towards UNC2 activation threshold. They also contribute to the late repolarization phase counterbalancing IRK, KQT3, EGL2 and KVS1 potassium currents, as stated by the faster repolarization compared to the WT case, both after the peak and at the stimulus removal.

As expected, KCNL deletion affects membrane repolarization after the peak and slightly alters the plateau phase (Fig 6F). Concerning IRK current, its suppression decreases the voltage peak due to reduced inward currents. Also, it significantly affects resting potential as observed also for NCA passive current (Fig 6A). The resting potential is equal to -74.4 mV in the wild type case, -84.1 mV in absence of NCA (Fig 6A), and -71.1 mV in absence of IRK current (Fig 6F). Therefore, we infer the significant contribution of inward rectifier and passive sodium channels in tuning the resting state. Also, NCA current deletion alters upstroke and plateau phases, in a way consistent with a delayed activation of active calcium and potassium currents induced by a lower resting potential. Finally, no significant changes are observed when suppressing SLO1 and SLO2 currents, possibly suggesting their minor role in the generation/modulation of the response (Fig 6E). It is worth mentioning that knockout neurons can not be directly compared with mutated neurons, because our model is missing the description of the plastic rearrangement of currents, occurring to balance the genetic lack of specific channels. However, *in silico* knockout models can shed light on the relative and cooperative roles of ion currents in the overall cell response.

#### RMD neuron

**Fig 7:**
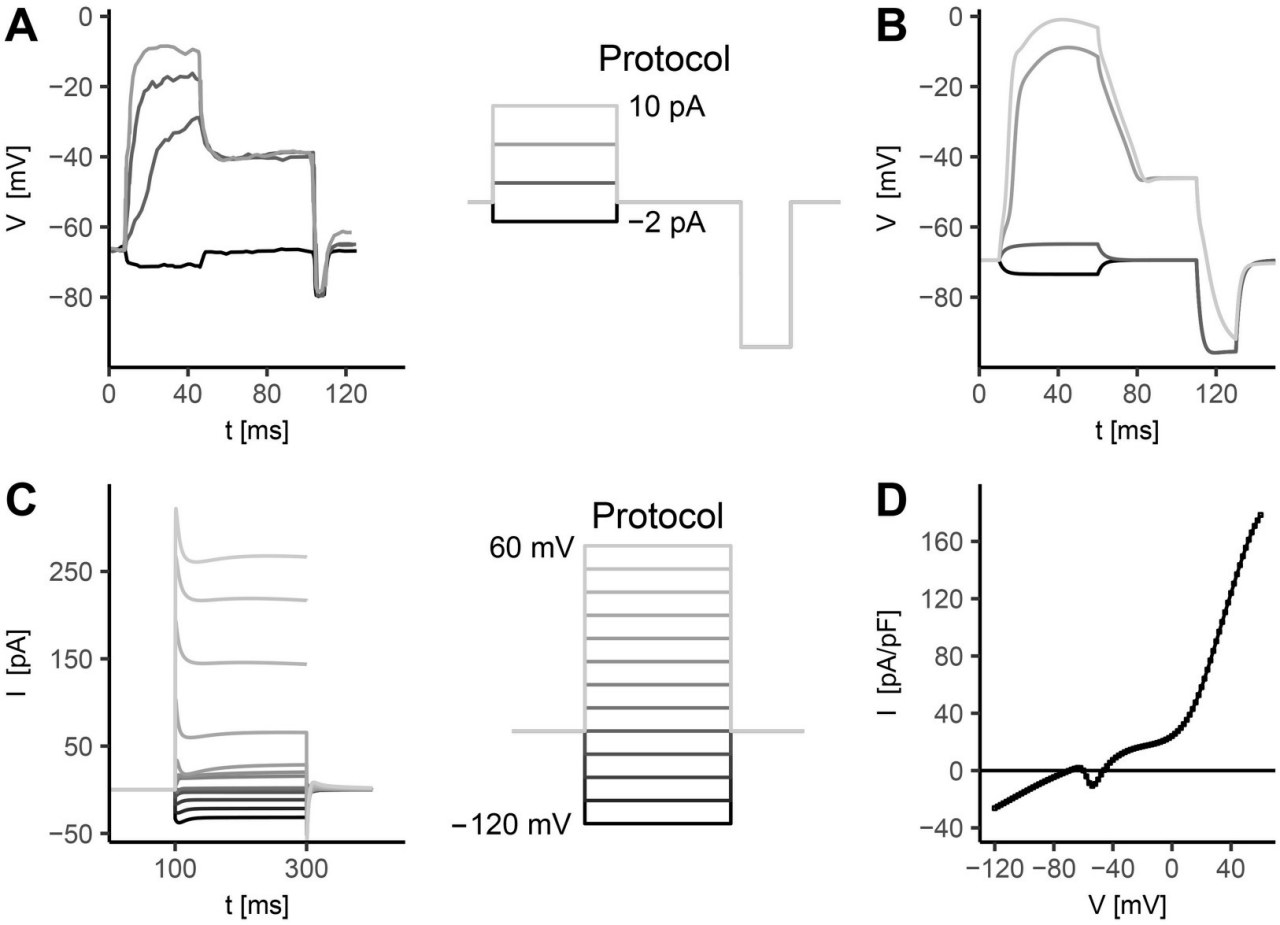
*In silico* RMD neuron response to voltage and current stimuli. **A) Experimental current clamp**. Experimental data from [13]. **B) Simulated current clamp for the RMD neuron**. The stimulation protocol, sketched in the middle column, consists in 4 current stimuli from -2 pA to 10 pA, followed by a negative step of 15 pA. The first step duration is 50 ms, while the duration of the negative step is 20 ms. The interval between the first and the second step is 50 ms. The holding current is *I*_*h*_ = 0pA. RMD shows a threshold-like response, characterized by large excursions of membrane voltage for *I*_ext_ > 6 pA, followed by a sustained plateau phase. A sensitivity analysis performed by varying the calcium removal rate *τ*_*Ca*_ (S7 Fig) does not show significant changes in the results. **C) Simulated voltage clamp for RMD neurons.** The stimulation protocol (sketched on the right) consists in a series of voltage steps from -120 mV to +60 mV with 15 mV increments. The holding potential is *V*_*h*_ = −70 mV and the step duration is 100 ms. **D) RMD steady-state I-V relation**. I-V curve is computed by averaging the current in the last 5 ms of the voltage step. The stimulation protocol consists in a series of 90 voltage steps between -120 mV and 60 mV. The step duration is 1200 ms and the holding potential is *V*_*h*_ = −70mV. The cell capacitance is set to 1.2 *μ*F to match membrane time constant as observed from experimental current clamp data [13], i.e. to fit the rise time of the membrane potential in response to current stimulation.

The RMD model is developed based on experimental data of current clamp [13] (Fig 7A).

In response to current pulses from 0 pA to 10 pA of 50 ms duration (Fig 7B), RMD shows a threshold-like response, characterized by large excursions of membrane voltage, similarly to AWC^ON^ (Fig 4C). After stimulus removal, the membrane potential stabilizes at a depolarized state (−46.6 mV), in agreement with experimental data [13]. This effect is further discussed below and it is related to an interesting bistable behaviour of RMD.

The RMD model is tested in voltage clamp conditions (Fig 7C). Similarly to AWC^ON^ (Fig 4A), in response to voltage pulses from a holding potential of -70 mV, membrane currents are mainly inward with passive behavior at command potentials *V* ≤ −70mV, and they show growing outward peaks at increasing potentials. Such peaks are followed by local minima before reaching the stationary value at intermediate voltage pulse intensities (between −60 mV and −30 mV). The steady-state I-V relation (Fig 7D) reveals a different behavior in RMD response compared to AWC^ON^ (Fig 4B), induced by the underlying combination of outward and inward components. Contrary to AWC^ON^, steady-state net current is negative at potentials below −69.5mV and between −59.8 mV and −46.6mV, while it becomes positive at increasing potentials. This multiple zero-crossing of the steady-state current significantly affects the neuron dynamics. Indeed, since the zero values of the I-V curve correspond to fixed points of the dynamical system, the computed I-V relation suggests that the modeled RMD has at least three stationary states.

**Fig 8:**
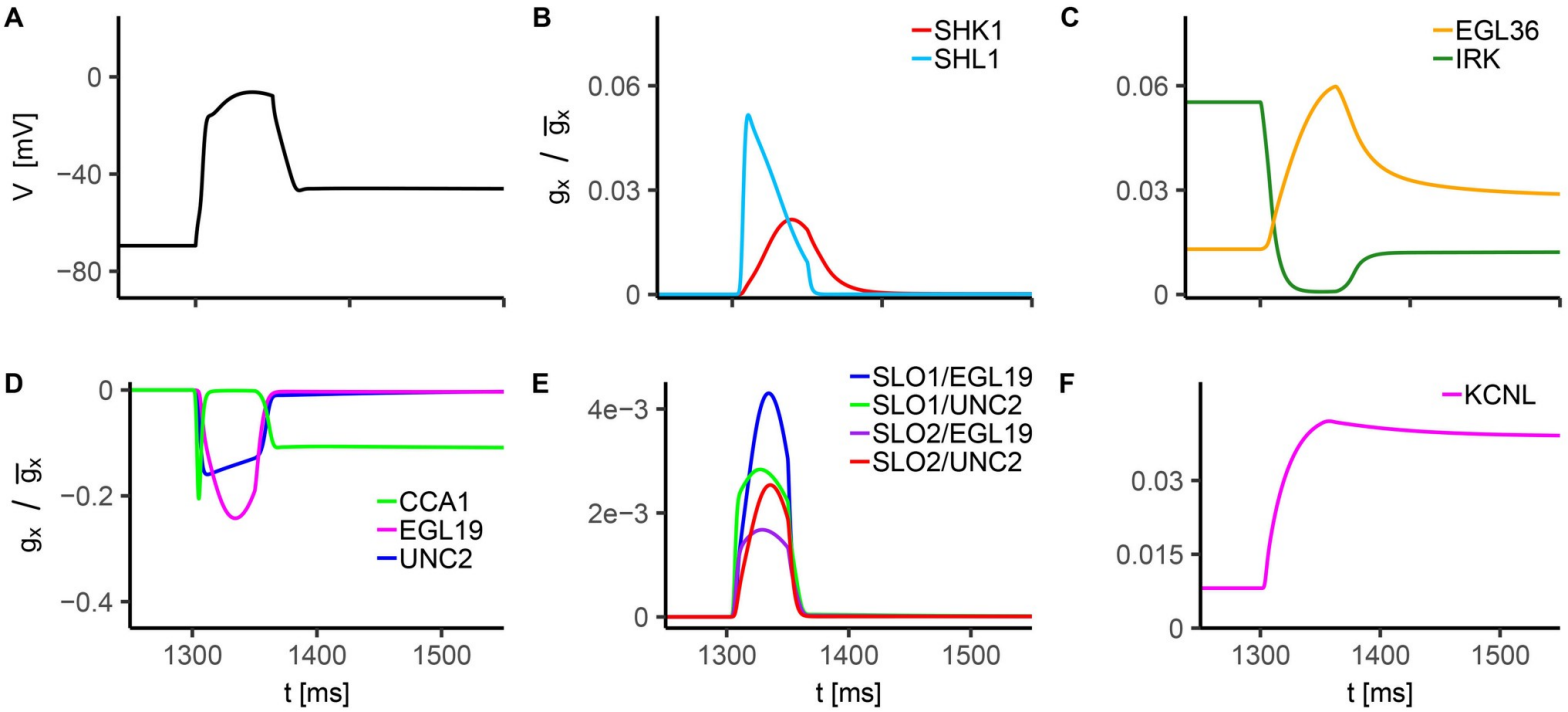
*In silico* analysis of RMD voltage response to current injection. The stimulation protocol consists in a single 10 pA current step with a duration of 50 ms. The holding current is *I*_*h*_ = 0 pA. **A) WT Voltage response to 10 pA current injection**. **B-F) Normalized conductance for modeled currents**. In these panels we report the time evolution of the normalized conductance of each modeled current for WT current clamp simulation shown in panel A. The normalized conductance is defined as the product of the activation and inactivation variables of the considered ion current. Panels B and C show $g_{x}/{\bar{g}}_{x}$ for voltage-gated K^+^ currents, panel D for voltage-gated Ca^2+^ currents, and panels E and F for calcium-dependent K^+^ currents. The $g/{\bar{g}}_{x}$ values for CCA1, UNC2 and EGL19 currents are multiplied by -1 to reproduce the sign of the associated currents.

**Fig 9:**
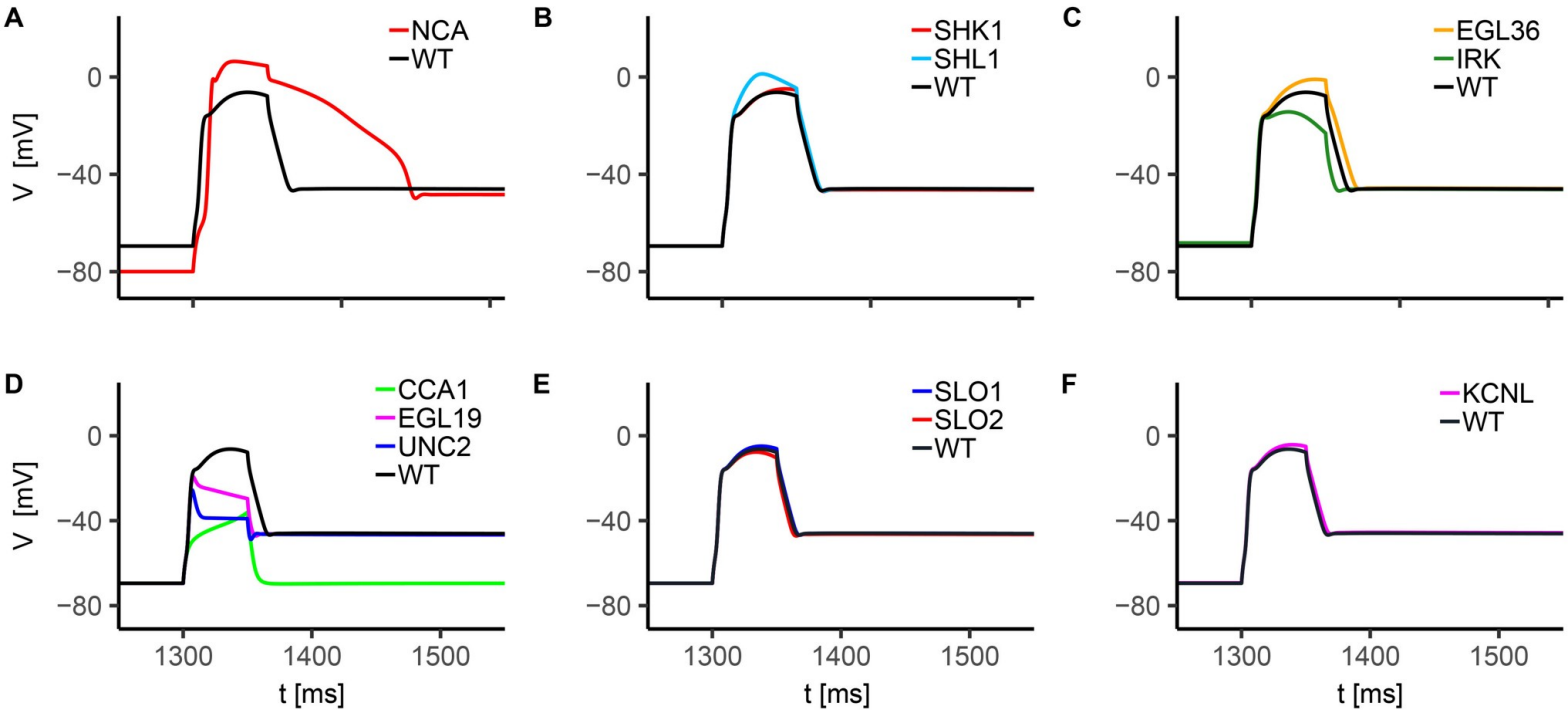
*In silico* RMD knockouts voltage response to current injection. The stimulation protocol consists in a single 10 pA current step with a duration of 50 ms. The holding current is *I*_*h*_ = 0 pA. Each knockout is obtained by suppressing the contribution of the desired current, leaving unchanged the other conductances. In panels A-F we report the response of NCA (A), voltage-gated potassium channels (B and C), voltage-gated calcium channels (D), and calcium-regulated potassium channels (E and F) knockouts.

We further analyze single currents activation and inactivation dynamics during current clamp (Fig 8A–8F), and build knockout neurons by selectively removing their contribution from the complete model (Fig 9A–9F).

WT simulation shows a significant activation of UNC2 and EGL19 currents, suggesting a leading role of these inward currents in the neuronal response (Fig 8D). In particular, their activation dynamics suggests that they affect the upstroke (mainly CCA1 and UNC2) and peak of membrane potential oscillation. The key role of UNC2 is therefore confirmed also in RMD [17, 37]. CCA1 current shows fast activation and inactivation within the early depolarization phase, and, interestingly, sustained activation during and after the repolarization phase. This behavior suggests a major contribution of the CCA-1 channels to the depolarized post-stimulus potential (Fig 8D), which is probably linked to their activation properties in response to voltage, i.e. they are low-voltage activated channels. From EGL36, SHK1 and SHL1 activation and inactivation patterns (Fig 8B and 8C), we infer that these outward currents shape the rising phase of membrane potential. In particular, SHL1 current affects the early depolarization phase, SHK1 the intermediate phase, and EGL36 the late depolarization and repolarization phases (Fig 8B and 8C). Interestingly, the late sustained activation of IRK, EGL36 and KCNL suggests that these potassium currents balance inward CCA1 current at the post-stimulus plateau (Fig 8D, 8C and 8F). In line with AWC^ON^, IRK current is significantly activated before the stimulus application and during the upstroke, suggesting a similar role in the regulation of electrical activity. Also, the low activation of SLO currents (Fig 8E) indicates a modest contribution to the action potential, in agreement with experimental findings for other motor neurons [14].

In the *in silico* knockout neurons, significant alterations of the response are caused by the suppression of CCA1, EGL19 and UNC2 (Fig 9D), confirming their role in neuron depolarization. Furthermore, the suppression of voltage sensitive potassium currents SHL1, SHK1 and EGL36 (Figs 9B and 8C) affects the early, middle and late phase of depolarization, respectively, corroborating also the role of these currents in shaping membrane potential oscillation, in agreement with the WT analysis. In accordance with AWC^ON^ (Fig 6E), SLO knockouts (Fig 9E) do not show significant alterations of the response, indicating a marginal contribution of these currents in RMD dynamics. The analysis of IRK and NCA knockouts (Fig 9A and 9C) further confirms the role of these currents in the regulation of resting potential. The most significant changes are caused by the suppression of NCA current, that leads to a decrease of the resting potential, from -69.5 mV to -80.0 mV. Additionally, NCA deletion affects membrane depolarization and repolarization. The same behavior but with smaller effects can be observed with IRK and KCNL suppression (Fig 9C and 9F). Strikingly, CCA1 current suppression leads to the disappearance of the post-stimulus depolarized potential induced by current injection, with the repolarization process recovering the original resting value of membrane potential (Fig 9D). Therefore, the depolarized state of the system at the post-stimulus plateau in WT seems to require a sustained inward current driven by CCA-1 channels to be supported.

#### Post-stimulus depolarization: Bistability analysis

**Fig 10:**
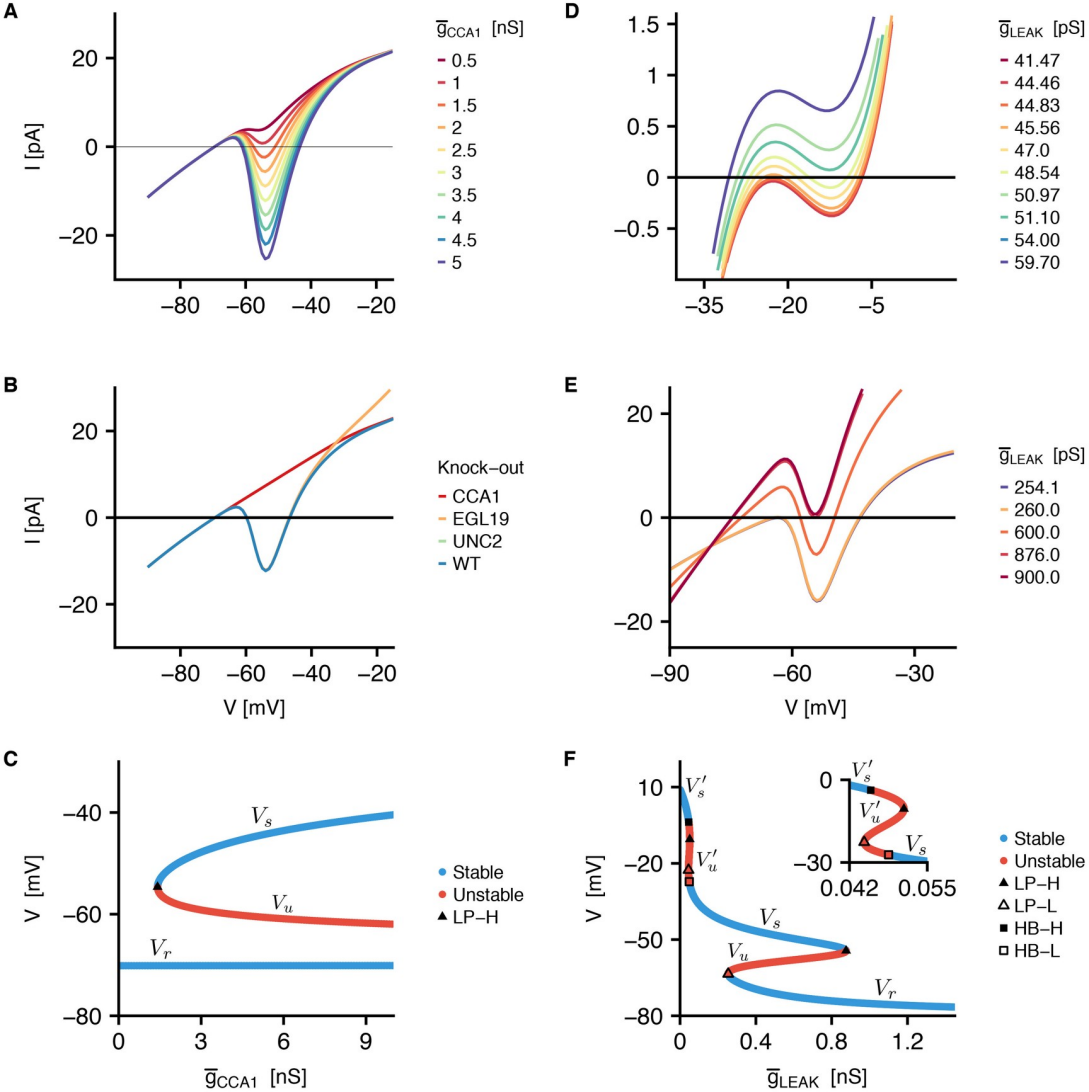
Analysis of the bistable behavior of RMD neuron. **A) Steady-state I-V curves at varying ${\bar{g}}_{\text{CCA}1}$.** Steady-state I-V curves are obtained from the currents computed during a voltage clamp stimulation with steps ranging between -90 mV and -10 mV, with 1 mV increments. The value of ${\bar{g}}_{\text{CCA}1}$ is varied between 0.5 nS and 5 nS with 0.5 nS increments. **B) Calcium channels knockouts steady-state I-V curves**. Voltage clamp simulation follows the same protocol of panel A. Knockout I-V curves are computed by removing the contribution of one of the CaV from the total current, leaving unchanged the other conductances. **C) Bifurcation diagram with ${\bar{g}}_{\text{CCA}1}$ as bifurcation parameter**. The black empty triangle represents the saddle-node (or limit point LP-H) bifurcation point [V, ${\bar{g}}_{\text{CCA}1}$] = [-59.5 mV, 1.14 nS]. At ${\bar{g}}_{\text{CCA}1}>1.14\;\text{nS}$ the system exhibits three fixed points: two stable (*V*_*r*_ and *V*_*s*_) and one unstable (*V*_*u*_). **D)-E) Steady-state I-V curves for different values of ${\bar{g}}_{\text{LEAK}}$**. The passive conductance is varied between 0.04 nS and 0.9 nS to obtain the steady-state I-V curve in correspondence of the different regimes highlighted also by the bifurcation diagram analysis (panel F). The applied voltage stimuli ranges from -90 mV to -30 mV with 0.8 mV increments, in panel D, and from -35 mV to +5 mV with 0.8 mV increments, in panel E. Step duration 1300 ms, holding potential *V*_*h*_ = −70 mV. **F) Bifurcation diagram with ${\bar{g}}_{\text{LEAK}}$ as bifurcation parameter**. For 0.25 nS $<{\bar{g}}_{\text{LEAK}}<0.9\;\text{nS}$ two stable solutions are separated by an unstable one. These correspond to *V*_*r*_, *V*_*s*_ and *V*_*s*_ in panel C. At lower values of ${\bar{g}}_{\text{LEAK}}$, and by decreasing the parameter, two unstable solutions $V_{u}^{\prime }$ and $V_{s}^{\prime }$ arise from a LP bifurcation (LP-H), with $V_{s}\leq V_{u}^{\prime }\leq V_{s}^{\prime }$. By decreasing the control parameter, $V_{s}^{\prime }$ gains stability through a subcritical Hopf bifurcation (HB-H), while at lower values of the control parameter, *V*_*s*_ loses stability through a second subcritical Hopf bifurcation (HB-L). Finally, by further lowering the parameter, $V_{u}^{\prime }$ and *V*_*s*_ collide in a second fold bifurcation (LP-L) and $V_{s}^{\prime }$ remains the only stable solution at even lower values of ${\bar{g}}_{\text{LEAK}}$. A second bistable regime may occur in between the two Hopf bifurcations.

The presence of two different stable resting potentials observed in RMD is related to the key role of CCA-1 channels. To completely characterize the role of these channels, we perform voltage clamp simulations at different values of CCA1 conductance ${\bar{g}}_{\text{CCA1}}$ (from 0.5 nS to 5 nS) (Fig 10A). For ${\bar{g}}_{\text{CCA1}}$ values above 1 nS, the steady-state current shows three different zeros, indicating three steady-states of the system. When ${\bar{g}}_{\text{CCA1}}$ decreases, the minimum of the I-V curve moves towards more positive values, with the consequent coalescence and disappearance of two zeros. We also analyzed the steady-state I-V curve in the range from -90 mV to -10 mV upon the suppression of EGL19, UNC2, and CCA1 currents (Fig 10B). It is clear that suppression of either EGL19 or UNC2 does not affect the curve significantly, confirming a key contribution of CCA1 current. Also, the simultaneous removal of various combinations of calcium currents (EGL19/UNC2, EGL19/CCA1, UNC2/CCA1, and EGL19/UNC2/CCA1) further confirms this result (S7 Fig). These observations all together suggest that CCA-1 channels are strictly connected to the presence of multiple steady-states in RMD neurons.

In order to characterize the steady-states stability and their variation upon CCA1 conductance modulation, we compute RMD equilibrium points by varying the parameter ${\bar{g}}_{\text{CCA1}}$. In the bifurcation diagram we report membrane potential values and the stability of the associated solutions as a function of CCA1 conductance (Fig 10C). For ${\bar{g}}_{\text{CCA1}}\leq 1.1\;\text{nS}$, there is only one stable state, corresponding to the resting state *V*_*r*_. At increasing values of ${\bar{g}}_{\text{CCA1}}(>1.1\;\text{nS})$, the system undergoes a saddle-node (fold or limit point LP in Fig 10C) bifurcation. Two solutions arise from this bifurcation, one stable (*V*_*s*_), and one unstable (*V*_*u*_), with *V*_*r*_ ≤ *V*_*u*_ ≤ *V*_*s*_. At high values of the CCA1 conductance, the unstable branch eventually collides with the lower stable resting state branch (*V*_*r*_) through a second LP bifurcation, leading to a sole high-voltage stable state. However, this regime is not of interest here, due to the non-physiological value of the parameter (with respect to the whole parameter set) and to the inconsistency of such unique high-voltage stable state with respect to experimental observations [13]. Remarkably, the unstable state *V*_*u*_ acts as a threshold, which regulates the switching of neuronal dynamics between the two stable states. Both in experiments [13] and in our simulations, current stimuli provide the required perturbations to induce such a switching, while pre- and post-stimulus resting potentials indicate the two different stable states.

Bistable dynamics is a prominent feature of various types of neurons, such as thalamic neurons, sensory neurons, Purkinje cells and motor neurons [10, 81–83]. Such bistable regimes involve the coexistence of different non-oscillatory stable states or different spiking modes, resting states and spiking, spiking and bursting. Detailed analyses also based on mathematical models [84–86] not only showed that calcium current is involved in neuron bistability, as we found in our analysis, but also highlighted the role of the leak current in such behavior. Thus, we further analyzed RMD bistable response by varying the leakage current conductance ${\bar{g}}_{\text{LEAK}}$ at fixed ${\bar{g}}_{\text{CCA1}}$.

In this case, steady-state current shows three different zeros at different ranges of the parameter, separated by a range of ${\bar{g}}_{\text{LEAK}}$ values at which the steady-state current presents a single zero crossing (Fig 10D and 10E). This suggests that modulations of ${\bar{g}}_{\text{LEAK}}$ can give rise to two different bistable regimes, as confirmed by the bifurcation diagram constructed by treating ${\bar{g}}_{\text{LEAK}}$ as control parameter, at fixed ${\bar{g}}_{\text{CCA1}}$ (Fig 10F). Two LP bifurcations give rise to a bistable regime when ${\bar{g}}_{\text{LEAK}}$ is in the range ∼0.25 nS −0.9 nS. Within this range two stable solutions are separated by one unstable solution. These solutions correspond to *V*_*r*_, *V*_*u*_ and *V*_*s*_ described previously. At values of ${\bar{g}}_{\text{LEAK}}$ lower than ∼0.25 nS a combination of two LP and two Hopf (HB) bifurcations can give rise to a second bisle regime, with two stable states *V*_*s*_ and $V_{s}^{\prime }$ separated by an unstable state $V_{u}^{\prime }(V_{s}\leq V_{u}^{\prime }\leq V_{s}^{\prime })$. Notably, the presence of Hopf bifurcations also suggests the occurrence of periodic solutions.

**Fig 11:**
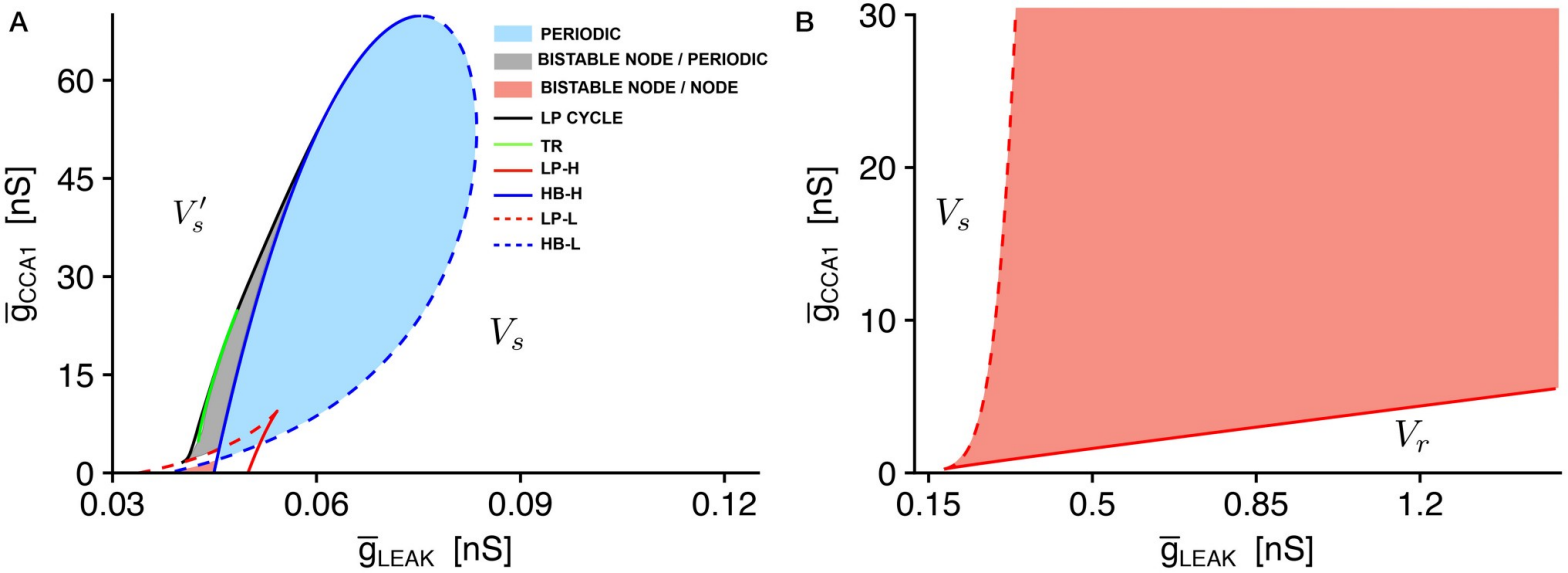
Bifurcation diagram in the plane ${\bar{g}}_{\text{LEAK}}-{\bar{g}}_{\text{CCA}1}$. Fold (LP) and Hopf (HB) bifurcation curves are highlighted in red and blue respectively, while black and green curves denote fold and torus bifurcations of cycles (LP cycle and TR, respectively), i.e., periodic solutions of the model. **A) Two-dimensional bifurcation diagram at low ${\bar{g}}_{\text{LEAK}}$ values**. At low values of ${\bar{g}}_{\text{CCA}1}$ we recover the one-dimensional bifurcation diagram described in Fig 10, and within the red area enclosed by HB-L and HB-H the system shows bistabilty, with *V*_*s*_ and $V_{s}^{\prime }$ as stable non-periodic states (nodes). At increasing values of ${\bar{g}}_{\text{CCA}1}$ two unstable cycles originate from a fold bifurcation of cycles (LP cycle) one of which collides with $V_{s}^{\prime }$ in the HB-H bifurcation at increasing values of ${\bar{g}}_{\text{LEAK}}$. In the very proximity of the fold bifurcation of cycles, the larger periodic solution gains stability via a torus bifurcation (TR). In between TR and HB-H bifurcations, the system shows a different kind of bistability, characterized by the coexistence of $V_{s}^{\prime }$ and a stable periodic solution (gray area). At higher values of ${\bar{g}}_{\text{LEAK}}$, on the right of HB-H, only the oscillatory solution survives (blue area), which eventually disappears through the HB-L bifurcation. White areas in the diagram denote the presence of a unique stable steady-state, $V_{s}^{\prime }$ and *V*_*s*_ at low and high values of ${\bar{g}}_{\text{LEAK}}$, respectively. **B) Two-dimensional bifurcation diagram at high ${\bar{g}}_{\text{LEAK}}$ values**. Below the LP bifurcation at low voltage (LP-L, red dashed line), there is a unique stable state *V*_*s*_, while after the bifurcation curve a second stable state *V*_*r*_ appears, together with an unstable state *V*_*u*_. Further increasing ${\bar{g}}_{\text{LEAK}}$, the stable state *V*_*s*_ coalesces with *V*_*u*_ in a second LP bifurcation at high voltage (LP-H, red continuous line), making the system collapse in the unique stable state *V*_*r*_. The bistability regime is guaranteed within the red area.

To grasp a complete picture of dynamical system solutions and bistability regimes, we further compute a two-dimensional bifurcation diagram summarizing model behavior in the plane ${\bar{g}}_{\text{LEAK}}-{\bar{g}}_{\text{CCA1}}$. Fig 11A focuses on the ${\bar{g}}_{\text{LEAK}}$ range 0.03nS −0.12nS. At low values of ${\bar{g}}_{\text{CCA1}}$ the system may show a single stable state ($V_{s}^{\prime }$ or *V*_*s*_) or the coexistence of such states if ${\bar{g}}_{\text{LEAK}}$ is within a suitable range (red area in Fig 11A). At increasing values of ${\bar{g}}_{\text{CCA1}}$ the presence of a stable periodic solution, originating from a torus bifurcation, adds additional complexity. In a narrow range of parameter values the stable solution $V_{s}^{\prime }$ coexists with a stable periodic solution (gray area in Fig 11A), which becomes the unique stable solution at values of ${\bar{g}}_{\text{LEAK}}$ approximately in the range 0.45 nS −0.8 nS (blue area in Fig 11A). A further increase of ${\bar{g}}_{\text{LEAK}}$ eventually suppresses such periodic solution making the system collapse on the unique stable state *V*_*s*_. Fig 11B shows a continuation of the two-dimensional diagram at values of ${\bar{g}}_{\text{LEAK}}$ ≥0.15 nS. In the region below the low LP bifurcation (red dashed line), the system exhibits the unique stable state *V*_*s*_, while above the bifurcation point the stable state *V*_*r*_ arises and the system comes back again in a bistable regime. At further increasing values of ${\bar{g}}_{\text{LEAK}}$, the second LP bifurcation eventually makes the system collapse in the unique stable state *V*_*r*_. The coexistence of the stable states *V*_*s*_ and *V*_*r*_ is guaranteed in a large region of the ${\bar{g}}_{\text{LEAK}}-{\bar{g}}_{\text{CCA1}}$ plane (red area). Notably, the one dimensional bifurcation diagrams presented in Fig 10C and 10F can be recovered as one-dimensional cuts on the two-dimensional bifurcation diagram, along ${\bar{g}}_{\text{LEAK}}$ or ${\bar{g}}_{\text{CCA1}}$ axis.

It is important to stress that the occurrence of periodic solutions supports the presence of oscillatory dynamics in membrane potential, i.e. action potentials. In our analysis, such action potentials are fired from a depolarized plateau of about -30 mV and show a peak and a period of ∼5 mV, and ∼200 ms, respectively. Interestingly, this is nicely in agreement with action potential features recently measured in AWA [10]. These periodic solutions indicate that our model can potentially replicate other electrical patterns experimentally recorded in *C. elegans* neurons. Dedicated analyses, involving an ad-hoc fitting of the model to the AWA case, would be required to investigate such behavior.

## Conclusions

Decades of studies have clarified many aspects of *C. elegans* neuronal physiology, but we still miss a comprehensive description of a huge number of biophysical mechanisms working both at the single cell and at the systemic level. In particular, the complex dynamics of its nervous system still has to be fully analyzed and understood, in spite of the *C. elegans* nervous system being one of the most studied neuronal circuitry, because of its relatively simple structure. The analysis of both spontaneous and evoked neuronal activity in this nematode could be crucial to investigate complex processes involved in sensory integration, information storage and transmission, and output control. In this regard, mathematical models play a fundamental role, not only in supporting experimental investigations, but more importantly in providing new insights of the studied phenomena.

In this paper, we report, for the first time to the best of our knowledge, a comprehensive biophysical framework for the modeling of *C. elegans* neurons, which includes the main voltage-gated, ligand-gated and voltage/ligand-gated ion channels [13, 15, 17, 19, 36, 57] expressed in a large variety of neuronal and muscle cells in this nematode.

Our biophysical model, starting from an accurate description of single ion currents, is able to capture the main features of AWC^ON^ and RMD neuronal dynamics [13, 59], selected as test cases.

AWC^ON^ voltage clamp simulations reproduce experimental whole-cell recordings [59], as confirmed also by comparison of steady-state I-V relations. We point out the ability of AWC neurons to produce regenerative responses triggered by the sequential activation of UNC-2 and EGL-19 calcium channels. Our analysis highlights the interplay between calcium UNC-2 and EGL-19 and potassium EGL-2 and KVS-1 channels, as experimentally observed in ASER neurons [57]. We also suggest a minor role of SLO-1 and SLO-2 channels in the generation/modulation of the response, similarly to VA5, VB6, and VD5 neurons [14].

Concerning RMD, our model properly reproduces current clamp experiments [13]. SHL-1, SHK-1 and EGL-36 channels have a leading role in RMD repolarization. Notably, and consistently with experimental whole-cell recordings [13], simulated RMD neurons show a peculiar behavior, characterized by the absence of full repolarization. We could asses the existence of two stable equilibrium states of the neuron dynamics (at about -70 mV and -47 mV), which exist in the absence of applied stimuli. Transient perturbations, in the form of current clamp, lead to a switching of RMD resting states. Notably, the RMD post-stimulus depolarized state is stable, while other *C. elegans* neurons classified as bistable (among which AFD) show full repolarization at the pre-stimulus resting state [10]. This may indicate that, in the absence of an applied current, such neurons have only one stable equilibrium, unlike RMD. We demonstrate that CCA-1 channels are directly involved in RMD bistability and their sustained activation supports the steady depolarization of the neuron. This is consistent with the disappearance of the depolarized equilibrium state after stimulus termination in CCA1 *in silico* knockout, as shown by experiments [13]

The differences found in the waveform of the potentials in AWC^ON^ and RMD may reflect the different functional role of these two neurons. AWC^ON^ is a sensory neuron, activated by chemical stimuli and characterized by a regenerative potential, eliciting active responses that outlast the stimulus pulse. AWC^ON^ activation is deterministic [79], directly activated by the cascade triggered by the odorant-receptor pairing. The AWC^ON^ graded potential reflects the shape and the duration of the stimulus without interference from other neurons. On the other hand, in the RMD motor neuron we observe a bistable potential, existing in either up and down states, and switched by the overall activity of upstream neurons. The flip-flop behavior is related to the regulation of the rhythmic movements of the nose and to the forward and backward locomotion [13]. Being a cell downstream of sensory neurons, RMD can be potentially affected by perturbations coming from the surrounding connectome. Therefore, the plateau potential and the bistability ensure robustness and resilience to noise by storing the sign of the most recent input [58].

Our model presents some approximations, whose strengths and limitations deserve to be discussed. Regarding the spatial extension of the system, we adopted a single compartment model. This approximation naturally discards diffusive currents induced by complex neuronal morphology as well as heterogeneities of mechanisms and ion conductances over dendritic, somatic and axon components. However, single compartment models are normally used to study electrophysiological dynamics in a large variety of cells and species. The obtained simulation results, in good agreement with experimental data, attest the validity of the single isopotential compartment approximation with particular regards to single cells studies [70, 87–89]. Concerning the calcium dynamics, we adopted a simplified description at the microscale, which does not include complex calcium fluxes within the cells. Although more detailed calcium models have been formulated [21–23], we chose to not include these complex descriptions in our model because of the different dynamical timescales analyzed.

With respect to the parameters determination, we selected specific parameter sets as calculated from least-squares and genetic algorithm optimization. Due to the high number of parameters included in our model, we cannot exclude that other parameter sets could be identified, reproducing experimental data with a similarly good degree of accuracy. New experiments, in particular for BK and SK channels, could be useful to uniquely constrain the parameter set in future studies. Nevertheless, our model is carefully built to include available data on *C. elegans* and it correctly reproduces electrophysiological behavior of ion channels and neuronal dynamics for selected neurons, i.e. AWC^ON^ and RMD.

Overall, we are confident that the above mentioned approximations do not affect significantly the descriptive and predictive power of our model. Indeed, the exploration of the dynamical system solutions, by changing the passive conductance values, shows the ability of the model to capture even the oscillatory behavior in membrane potential, as experimentally observed in AWA neurons [10]. We therefore expect that our model should be potentially used also to reproduce all-or-none action potential or bursting like oscillations, allowing to deeply investigate the phenomenon and clarify its underlying molecular mechanisms.

In conclusion,the modeling framework here presented could be a fundamental tool to investigate *C. elegans* neuronal responses, both at the single cell and at the cellular network level, with important implications for a comprehensive understanding of nervous system functions.

## Supporting information

S1 File Model equations. List of equations used to model the channel currents.
(PDF)

S1 Table Model parameters. Fitted parameters, as identified by least-squares nonlinear fitting (or by genetic algorithm in the case of SLO channels, see Methods) are reported. Corresponding steady-state activation/inactivation and time constant functions, computed with the fitted parameters, are shown in S1–S5 Figs. We refer the reader to the modeled equations (Eqs 12–19, 26–29, and A-C in S1 File) explicitly including the fitted parameters. Some channel contributions (SHL1, KVS1, KQT3, EGL2, EGL19, UNC2, CCA1) were modeled based on homologous channels in species different from *C. elegans* or on muscle cells. To include these contributions in the entire neuron model, we *a posteriori* calibrated some of the fitted parameters to match voltage clamp and current clamp data [13, 59]. We shifted half-activation and inactivation potentials and time constants. Such calibrated parameters are reported in parentheses.
(PDF)

S1 FigSHL1, KVS1, and KQT3 currents steady-state activation/inactivation variables and time constants.In panels A-C we report the steady-state activation and inactivation curves (left), the activation time constant function (center), and the inactivation time constant function (right). **A) SHL1 currents**. On the left we report steady-state activation (red) and inactivation (blue) functions (Eqs A1 and A3 in S1 File). In the middle the activation time constant function ($\tau_{m_{\text{SHL1}}}^{\text{s}}$, Eq A2 in S1 File) is represented together with the fitted experimental values (black dots, from [25]). In the right panel the solid lines represent the slow inactivation time constant ($\tau_{h_{\text{SHL1}}}^{\text{s}}$, Eq A4 in S1 File), while the dashed line describes the fast one ($\tau_{h_{\text{SHL1}}}^{\text{f}}$, Eq A4 in S1 File). In this case, black dots are the experimental points extracted from [25]. **B) KVS1 currents**. Steady-state activation (red) and inactivation (blue), with experimental points (blue and red dots, from [31]), are represented on the left (Eqs A6 and A7 in S1 File). Middle and right panels show respectively activation and inactivation time constants as function of voltage (Eq A8 in S1 File, [31]). **C) KQT3 currents**. Steady-state activation (red, Eq A15 in S1 File, from [26]) and inactivation (blue and green, Eq A18 in S1 File, from [73]) variables are shown on the left. The red dots are experimental points from [26]. Middle panel shows fast (dashed) and slow (solid) activation time constants (Eqs A16 and A17 in S1 File, from [73]). On the left the inactivation time constant ($\tau_{w_{\text{KQT}3}}$, Eq A19 in S1 File) is represented.(TIF)

S2 Fig SHK1, EGL2, EGL36, and IRK currents steady-state activation/inactivation variables and time constants. In panels A-D we report the steady-state activation and inactivation curves (left), and the activation time constant function (right). **A) SHK1 currents**. Steady-state activation (red, Eq A10 in S1 File) and inactivation (blue, Eq A12 in S1 File) functions are represented on the left. Blue and red dots are the experimental points from [25]. On the right the activation time constant function is shown (Eq A11 in S1 File) with fitted experimental points (black dots) from [29]. **B) EGL2 currents**. On the left is represented the steady-state activation variable (Eq A22 in S1 File) with experimental data (red dots) from [28]. On the left the activation time constant function (Eq A23 in S1 File) is shown. **C) EGL36 currents**. Steady-state activation variable (Eq A25 in S1 File) is shown on the left, with experimental data from [30] (red dots). Fast (red), medium (green) and slow (blue) activation time constants (Eq A26 in S1 File) are shown on the right. Red, green and blue dots represent experimental measurements as reported in [30]. **D) IRK currents**. Steady-state activation variable is represented on the right (Eq A28 in S1 File). On the left the activation time constant (Eq A29 in S1 File) is represented, with experimental data (black dots) from [35].
(TIF)

S3 Fig EGL19, UNC2, and CCA1 currents steady-state activation/inactivation variables and time constants. In panels A-C we report the steady-state activation and inactivation curves (left), the activation time constant function (center), and the inactivation time constant function (right). **A) EGL19 currents**. Steady-state activation (red, Eq B1 in S1 File) and inactivation (blue, Eq B3 in S1 File) functions are plotted in the left panel together with the experimental data (blue and red dots, from [15]). In the middle and right panels the activation (Eq B12 in S1 File, experimental data from [36]) and inactivation (Eq B4 in S1 File, experimental data from [36]) time constant functions are represented, respectively. **B) UNC2 currents**. In the right panel the steady-state activation (red, Eq B6 in S1 File) and inactivation (blue, Eq B8 in S1 File) functions are shown. Red and blue dots denote experimental data taken from [37]. Activation time constant (Eq B7 in S1 File) together with experimental data from [38] (black dots) is shown in the middle panel, while inactivation time constant (Eq B9 in S1 File) with experimental data from [38] is represented in the right panel. **C) CCA1 currents**. Steady-state activation (Eq B11 in S1 File) and inactivation variables (Eq B12 in S1 File) are shown on the left, together with experimental data from [40] (red and blue dots). Middle and left panels show activation and inactivation time constants (Eq B14 in S1 File) with experimental data (black dots) from [40].
(TIF)

S4 FigIsolated SLO1 and SLO2 currents activation variables and time constants.**A) Genetic algorithm fitting results for SLO1 isolated currents**. Best fitting results obtained with the genetic algorithm for isolated (uncoupled from CaV) SLO1 currents at different calcium concentrations: steady-state activation (left, Eq 16) and time constant (right, Eq 19) functions. Experimental data (colored dots) from [41]. **B) Genetic algorithm fitting results for SLO2 isolated currents**. Best fitting results obtained with the genetic algorithm for isolated SLO2 currents at different calcium concentrations: steady-state activation (left, Eq 16) and time constant (right, Eq 19) functions. Experimental data (colored dots) from [42]. **C) Simulation of isolated SLO1 currents**. Isolated SLO1 currents (Eq 20) in response to voltage stimuli between -70 mV and 40 mV for ${\left[{\text{Ca}}^{2+}\right]}_{i}^{n}=1,\;10,\;100,\;100\;\mu \;\text{M}$. **D) Simulation of isolated SLO2 currents**. Isolated SLO2 currents (Eq 20) in response to voltage stimuli between -70 mV and 40 mV for ${\left[{\text{Ca}}^{2+}\right]}_{i}^{n}=1,\;10,\;100,\;100\;\mu \;\text{M}$. In panels C and D the voltage step duration is 300 ms and the holding potential is *V*_*h*_ = −80mV. The channel conductances (${\bar{g}}_{\text{BK}}$) are assumed both equal to 15 nS.(TIF)

S5 FigKCNL currents.KCNL currents steady-state activation (Eq C6 in S1 File) as function of the intracellular calcium concentration ${\left[{\text{Ca}}^{2+}\right]}_{i}^{m}$.(TIF)

S6 Fig AWC^ON^ neuron simulation. **A) Steady-state I-V curve computed in current clamp**. The stimulation protocol consists in multiple current steps ranging from -30 pA to 30 pA. The step duration is 1000 ms and the holding current is *I*_*h*_ = 0 pA. V-I curve is calculated by averaging the membrane voltage in the last 5 ms of the current step. **B) Calcium sensitivity analysis**. Effects of *τ*_*Ca*_ (Eq 28) changes on membrane voltage response to current stimuli. The stimulation protocol consists in a current step of 15 pA with a duration of 1300 ms, the holding current *I*_*h*_ = 0 pA. For clarity, in this panel we show an enlargement of the repolarization phase of the response, since the slight variation of the response can mostly be observed within this phase.
(TIF)

S7 Fig RMD neuron simulation. **A) Steady-state I-V curve for double Ca^2+^ channels knockout**. I-V curves are calculated by averaging the current in the last 5 ms of each voltage step. The voltage clamp protocol consists in multiple voltage steps between -90 mV and -10 mV. The step duration is 1300 ms and the holding potential is *V*_*h*_ = −70 mV. **B) RMD neuron calcium sensitivity analysis.** Simulated response of RMD neuron at different values of *τ*_*Ca*_ (Eq 28). The stimulation protocol consists in a 10 pA current step with a duration of 50 ms (holding current *I*_*h*_ = 0 pA). For clarity, in this panel we show an enlargement of the repolarization phase of the response, since the slight variation of the response can mostly be observed within this phase.
(TIF)
