# Correction: Biophysical modeling of *C*. *elegans* neurons: Single ion currents and whole-cell dynamics of AWC^on^ and RMD

**DOI:** 10.1371/journal.pone.0256930

**Published:** 2021-08-26

**Authors:** Martina Nicoletti, Alessando Loppini, Letizia Chiodo, Viola Folli, Giancarlo Ruocco, Simonetta Filippi

In the Channel modeling subsection of the Methods, there is an error in the fourth equation. There should be a plus (+) sign between the 1 and the exponential. The correct equation is:

$$m_{x,\infty }(V)=\frac{1}{1+e^{\frac{-(V-V_{0.5})}{k_{a}}}}$$

There are multiple errors in S1 File. There are errors in equations A1, A6, A10, A15, A22, A25, A28, B1, B3, B6, B11, and B13. Please view the correct S1 File below.

There are multiple errors in S1 Table. Please view the correct S1 Table below.

There are multiple errors in S1 Fig. The left panel of row A, the right panel of row B, and several panels of row C are incorrect. Please view the correct S1 Fig below.

There is an error in S2 Fig. The right panel of row B is incorrect. Please view the correct S2 Fig below.

## Supporting information

S1 File Model equations. List of equations used to model the channel currents.
(PDF)

S1 Table Model parameters. Fitted parameters, as identified by least-squares nonlinear fitting (or by genetic algorithm in the case of SLO channels, see Methods) are reported. Corresponding steady-state activation/inactivation and time constant functions, computed with the fitted parameters, are shown in S1–S5 Figs. We refer the reader to the modeled equations (Eqs 12–19, 26–29, and A-C in S1 File) explicitly including the fitted parameters. Some channel contributions (SHL1, KVS1, KQT3, EGL2, EGL19, UNC2, CCA1) were modeled based on homologous channels in species different from *C*. *elegans* or on muscle cells. To include these contributions in the entire neuron model, we *a posteriori* calibrated some of the fitted parameters to match voltage clamp and current clamp data [13, 59]. We shifted half-activation and inactivation potentials and time constants. Such calibrated parameters are reported in parentheses.
(PDF)

S1 FigSHL1, KVS1, and KQT3 currents steady-state activation/inactivation variables and time constants.In panels A-C we report the steady-state activation and inactivation curves (left), the activation time constant function (center), and the inactivation time constant function (right). **A) SHL1 currents**. On the left we report steady-state activation (red) and inactivation (blue) functions (Eqs A1 and A3 in S1 File). In the middle the activation time constant function ($\tau_{m_{SHL1}}^{S}$, Eq A2 in S1 File) is represented together with the fitted experimental values (black dots, from [25]). In the right panel the solid lines represent the slow inactivation time constant ($\tau_{h_{SHL1}}^{S}$, Eq A4 in S1 File), while the dashed line describes the fast one ($\tau_{h_{SHL1}}^{f}$, Eq A4 in S1 File). In this case, black dots are the experimental points extracted from [25]. **B) KVS1 currents**. Steady-state activation (red) and inactivation (blue), with experimental points (blue and red dots, from [31]), are represented on the left (Eqs A6 and A7 in S1 File). Middle and right panels show respectively activation and inactivation time constants as function of voltage (Eq A8 in S1 File, [31]). **C) KQT3 currents**. Steady-state activation (red, Eq A15 in S1 File, from [26]) and inactivation (blue and green, Eq A18 in S1 File, from [73]) variables are shown on the left. The red dots are experimental points from [26]. Middle panel shows fast (dashed) and slow (solid) activation time constants (Eqs A16 and A17 in S1 File, from [73]). On the left the inactivation time constant ($\tau_{w_{KQT3}}$, Eq A19 in S1 File) is represented.(PDF)

S2 Fig SHK1, EGL2, EGL36, and IRK currents steady-state activation/inactivation variables and time constants. In panels A-D we report the steady-state activation and inactivation curves (left), and the activation time constant function (right). **A) SHK1 currents**. Steady-state activation (red, Eq A10 in S1 File) and inactivation (blue, Eq A12 in S1 File) functions are represented on the left. Blue and red dots are the experimental points from [25]. On the right the activation time constant function is shown (Eq A11 in S1 File) with fitted experimental points (black dots) from [29]. **B) EGL2 currents**. On the left is represented the steady-state activation variable (Eq A22 in S1 File) with experimental data (red dots) from [28]. On the left the activation time constant function (Eq A23 in S1 File) is shown. **C) EGL36 currents**. Steady-state activation variable (Eq A25 in S1 File) is shown on the left, with experimental data from [30] (red dots). Fast (red), medium (green) and slow (blue) activation time constants (Eq A26 in S1 File) are shown on the right. Red, green and blue dots represent experimental measurements as reported in [30]. **D) IRK currents**. Steady-state activation variable is represented on the right (Eq A28 in S1 File). On the left the activation time constant (Eq A29 in S1 File) is represented, with experimental data (black dots) from [35].
(PDF)
