# Supplementary material for: Biophysical modeling of C. elegans neurons: Single ion currents and whole-cell dynamics of AWCon and RMD
Source: PLoS One. 2019 Jul 1;14(7):e0218738. doi: 10.1371/journal.pone.0218738 (PMC6602206; doi:10.1371/journal.pone.0218738)
Supplement: S1 Table — Fitted parameters, as identified by least-squares nonlinear fitting (or by genetic algorithm in the case of SLO channels, see Methods) are reported. Corresponding steady-state activation/inactivation and time constant functions, computed with the fitted parameters, are shown in S1–S5 Figs. We refer the reader to the modeled equations (Eqs 12–19, 26–29, and A-C in S1 File) explicitly including the fitted parameters. Some channel contributions (SHL1, KVS1, KQT3, EGL2, EGL19, UNC2, CCA1) were modeled based on homologous channels in species different from C. elegans or on muscle cells. To include these contributions in the entire neuron model, we a posteriori calibrated some of the fitted parameters to match voltage clamp and current clamp data [13, 59]. We shifted half-activation and inactivation potentials and time constants. Such calibrated parameters are reported in parentheses. (PDF) [file pone.0218738.s002.pdf]

S1 TABLE. MODEL PARAMETERS

**Voltage-gated K$^+$ currents**			
**SHL1**			
**Parameter**		**Value**	**Unit**
m_∞	$V_{0.5}$	11.2 (-6.8)	mV
	k_a	14.1	mV
h_∞	$V_{0.5}$	-33.1	mV
	k_i	8.3	mV
τ_m	a	13.8 (1.4)	ms
	b	-17.5	mV
	c	12.9	mV
	d	-3.7	mV
	e	6.5	mV
	f	1.9 (0.2)	ms
τ_h^{f}	a	539.2 (53.9)	ms
	b	-28.2	mV
	c	4.9	mV
	d	27.3 (2.7)	ms
τ_h^{s}	a	8422.0 (842.2)	ms
	b	-37.7	mV
	c	6.4	mV
	d	118.9 (11.9)	ms
**SHK1**			
m_∞	$V_{0.5}$	20.4	mV
	k_a	7.7	mV
h_∞	$V_{0.5}$	-7.0	mV
	k_i	5.8	mV
τ_m	a	26.6	ms
	b	-33.7	mV
	c	15.8	mV
	d	-33.7	mV
	e	11.2	mV
	f	3.8	ms
τ_h	a	1400	ms
**KVS1**			
m_∞	$V_{0.5}$	57.1 (27.1)	mV
	k_a	25.0	mV
h_∞	$V_{0.5}$	47.3 (17.3)	mV
	k_i	11.1	mV
τ_m	a	30.0 (3.0)	ms
	b	18.1	mV
	c	-20	mV
	d	1.0 (0.1)	ms

τ_h^{f}	a	88.5 (8.9)	ms
	b	50.0	mV
	c	-15.0	mV
	d	53.4 (5.3)	ms
**KQT3**			
m_∞	$V_{0.5}$	-12.8 (7.7)	mV
	k_a	15.8	mV
w_∞	$V_{0.5}$	-1.1	mV
	k_i	28.8	mV
	a	0.5	
	b	0.5	
s_∞	$V_{0.5}$	-45.3	mV
	k_i	12.3	mV
	a	0.3	
	b	0.7	
τ_m^{f}	a	395.3 (39.5)	ms
	b	-38.1	mV
	c	33.6	mV
τ_m^{s}	a	5503.0 (550.3)	ms
	b	5345.4 (534.5)	ms
	c	-0.0283	$\mathrm{mV^{-1}}$
	d	-23.9	mV
	e	4590 (459.1)	ms
	f	-0.0357	$\mathrm{mV^{-1}}$
	g	14.2	mV
τ_w	a	0.5	ms
	b	2.9	ms
	c	-48.1	mV
	d	48.8	mV
τ_s	a	500	ms
**EGL2**			
m_∞	$V_{0.5}$	6.9	mV
	k_a	14.9	mV
τ_m	a	16.8 (8.4)	ms
	b	-122.6	mV
	c	-13.8	mV
	d	8.1 (4.1)	ms
**EGL36**			
m_∞	$V_{0.5}$	63.0	mV
	k_a	28.5	mV
τ_m^{s}	a	355.0	ms
τ_m^{m}	a	63.0	ms
τ_m^{f}	a	13.0	ms
**IRK**			
m_∞	$V_{0.5}$	-86.5	mV
	k_a	-28.0	mV
τ_m	a	17.1	ms

	b	-17.8	mV
	c	20.3	mV
	d	-43.4	mV
	e	11.2	mV
	f	3.8	ms
**Voltage-gated Ca^{2+} currents**			
**EGL19**			
m_∞	$V_{0.5}$	5.6 (-4.4)	mV
	k_a	7.5	mV
h_∞	$V_{0.5}$	24.9 (14.9)	mV
	k_i	12.0	mV
	k_i^b	-10.5 (-20.5)	mV
	$V_{0.5}^b$	8.1	mV
	a	1.4	
	b	0.1	
	c	6.0	
	d	0.6	
τ_m	a	2.9	ms
	b	5.2 (-4.8)	mV
	c	6.0	mV
	d	1.9	ms
	e	1.4 (-8.6)	mV
	f	30.0	mV
	g	2.3	ms
τ_h	a	0.4	
	b	44.6	ms
	c	-23.0 (-33.0)	mV
	d	5.0	mV
	e	36.4	ms
	f	28.7(18.7)	mV
	g	3.7	mV
	h	43.1	ms
**UNC2**			
m_∞	$V_{0.5}$	-12.2 (-37.2)	mV
	k_a	4.0	mV
h_∞	$V_{0.5}$	-52.5 (-77.5)	mV
	k_i	5.6	mV
τ_m	a	4.5	ms
	b	-8.2 (-38.2)	mV
	c	9.1	mV
	d	15.4	mV
	e	0.3	ms
τ_h	a	83.8 (142.5)	ms
	b	52.9 (22.9)	mV
	c	3.5	mV
	d	72.1 (122.6)	ms
	e	23.9 (-6.1)	mV
	f	3.6	mV

**CCA1**			
m_∞	$V_{0.5}$	-43.32(-57.7)	mV
	k_a	7.6 (2.4)	mV
h_∞	$V_{0.5}$	-58.0 (-73.0)	mV
	k_i	7.0 (8.1)	mV
τ_m	a	40.0 (20)	ms
	b	-62.5 (-92.5)	mV
	c	-12.6 (21.1)	mV
	d	0.7 (0.4)	ms
τ_h	a	280 (22.4)	ms
	b	-60.7 (-75.7)	mV
	c	8.5 (9.4)	mV
	d	19.8 (1.6)	ms
**Ca^{2+}-regulated K^+ currents**			
**SLO1**			
w_{yx}		0.013	mV^{-1}
w_{xy}		-0.028	mV^{-1}
w_0^-		3.15	ms^{-1}
w_0^+		0.16	ms^{-1}
K_{xy}		55.73	μM
n_{xy}		1.30	
K_{yx}		0.034	μM
n_{yx}		10^{-4}	
**SLO2**			
w_{yx}		0.019	mV^{-1}
w_{xy}		-0.024	mV^{-1}
w_0^-		0.87	ms^{-1}
w_0^+		0.028	ms^{-1}
K_{xy}		93.45	μM
n_{xy}		1.84	
K_{yx}		3294.55	μM
n_{yx}		10^{-5}	
**KCNL**			
K_{Ca}		0.33	μM
τ_m	a	6.3	ms
**Intracellular calcium calculation**			
g_{sc}		40	pS
V_{Ca}		60	mV
r		13	nm
F		96485	$C\,mol^{-1}$
D_{Ca}		250	$\mu^2 m\,s^{-1}$
k_B^+		500	$\mu M^{-1}\,s^{-1}$
$[B]_{\text{tot}}$		30	μM
$[\text{Ca}^{2+}]_{c,i}^n$		0.05	μM
V_{cell}		31.16 (AWC), 5.65 (RMD)	μm^3
f		0.001	
τ_{Ca}		50	ms

$[Ca^{2+}]_{eq}^{m}$	0.05	μM
