# Supplementary material for: Correction: Biophysical modeling of C. elegans neurons: Single ion currents and whole-cell dynamics of AWCon and RMD
Source: PLoS One. 2021 Aug 26;16(8):e0256930. doi: 10.1371/journal.pone.0256930 (PMC8389473; doi:10.1371/journal.pone.0256930)
Supplement: S1 File — List of equations used to model the channel currents. (PDF) [file pone.0256930.s001.pdf]

S1 FILE. MODEL EQUATIONS

For each modeled channel x, equations for steady-state activation and inactivation curves, time constants and current are reported below. When necessary, m_x^{f}, m_x^{m}, m_x^{s} (h_x^{f}, h_x^{m}, h_x^{s}) denote fast, medium and slow component, respectively, of activating (inactivating) variable associated to the x channel, while $\tau_{m_x}^{\mathrm{f}}$, $\tau_{m_x}^{\mathrm{m}}$, $\tau_{m_x}^{\mathrm{s}}$ ($\tau_{h_x}^{\mathrm{f}}$, $\tau_{h_x}^{\mathrm{m}}$, $\tau_{h_x}^{\mathrm{s}}$) denote the corresponding time constants. Data obtained from these equations are reported in the corresponding panels of Figs 1, 2, 3, and S1, S2, S3, S5 Figs. Fitted values of parameters are reported in S1 Table.

A. Voltage-gated potassium currents

SHL1

(A1) $$m_{\mathrm{SHL1},\infty}(V) = \frac{1}{1+e^{\frac{-(V-V_{0.5})}{k_a}}}$$

(A2) $$\tau_{m_{\mathrm{SHL1}}}(V) = \frac{a}{e^{\frac{-(V-b)}{c}} + e^{\frac{(V-d)}{\tilde{e}}}} + f$$

(A3) $$h^{\mathrm{f}}_{\mathrm{SHL1},\infty}(V) = h^{\mathrm{s}}_{\mathrm{SHL1},\infty}(V) = \frac{1}{1+e^{\frac{(V-V_{0.5})}{k_i}}}$$

(A4) $$\tau^{\mathrm{f}}_{h_{\mathrm{SHL1}}}(V) = \tau^{\mathrm{s}}_{h_{\mathrm{SHL1}}}(V) = \frac{a}{1+e^{\frac{(V-b)}{c}}} + d$$

(A5) $$I_{\mathrm{SHL1}} = \overline{g}_{\mathrm{SHL1}} \cdot m^3_{\mathrm{SHL1}} \cdot (0.7\, h^{\mathrm{f}}_{\mathrm{SHL1}} + 0.3\, h^{\mathrm{s}}_{\mathrm{SHL1}}) \cdot (V - V_K)$$

KVS1

(A6) $$m_{\mathrm{KVS1},\infty}(V) = \frac{1}{1+e^{\frac{-(V-V_{0.5})}{k_a}}}$$

(A7) $$h_{\mathrm{KVS1},\infty}(V) = \frac{1}{1+e^{\frac{(V-V_{0.5})}{k_i}}}$$

(A8) $$\tau_{m_{\mathrm{KVS1}}}(V) = \tau_{h_{\mathrm{KVS1}}}(V) = \frac{a}{1+e^{\frac{(V-b)}{c}}} + d$$

(A9) $$I_{\mathrm{KVS1}} = \overline{g}_{\mathrm{KVS1}} \cdot m_{\mathrm{KVS1}} \cdot h_{\mathrm{KVS1}} \cdot (V - V_K)$$

SHK1

(A10) $$m_{\mathrm{SHK1},\infty}(V) = \frac{1}{1+e^{\frac{-(V-V_{0.5})}{k_a}}}$$

(A11) $$\tau_{m_{\mathrm{SHK1}}}(V) = \frac{a}{e^{\frac{-(V-b)}{c}} + e^{\frac{(V-d)}{\tilde{e}}}} + f$$

(A12) $$h_{\mathrm{SHK1},\infty}(V) = \frac{1}{1+e^{\frac{(V-V_{0.5})}{k_i}}}$$

(A13) $$\tau_{h_{\mathrm{SHK1}}} = a$$

(A14) $$I_{\mathrm{SHK1}} = \overline{g}_{SHK1} \cdot m_{\mathrm{SHK1}} \cdot h_{\mathrm{SHK1}} \cdot (V - V_K)$$

KQT3

$$m^{\mathrm{f}}_{\mathrm{KQT3},\infty}(V) = m^{\mathrm{s}}_{\mathrm{KQT3},\infty}(V) = \frac{1}{1+e^{\frac{-(V-V_{0.5})}{k_a}}} \tag{A15}$$

$$\tau^{\mathrm{f}}_{m_{\mathrm{KQT3}}}(V) = \frac{a}{1+\left(\frac{V+b}{c}\right)^2} \tag{A16}$$

$$\tau^{\mathrm{s}}_{m_{\mathrm{KQT3}}}(V) = a + \frac{b}{1+10^{-c(d-V)}} + \frac{\tilde{e}}{1+10^{-f(g+V)}} \tag{A17}$$

$$w_{\mathrm{KQT3},\infty}(V) = s_{\mathrm{KQT3},\infty}(V) = a + \frac{b}{1+e^{\frac{(V-V_{0.5})}{k_i}}} \tag{A18}$$

$$\tau_{w_{\mathrm{KQT3}}}(V) = a + \frac{b}{1+\left(\frac{V-c}{d}\right)^2} \tag{A19}$$

$$\tau_{s_{\mathrm{KQT3}}} = a \tag{A20}$$

$$I_{\mathrm{KQT3}} = \overline{g}_{\mathrm{KQT3}} \cdot (0.7\, m^{\mathrm{f}}_{\mathrm{KQT3}} + 0.3\, m^{\mathrm{s}}_{\mathrm{KQT3}}) \cdot w_{\mathrm{KQT3}} \cdot s_{\mathrm{KQT3}} \cdot (V - V_K) \tag{A21}$$

EGL2

$$m_{\mathrm{EGL2},\infty}(V) = \frac{1}{1+e^{\frac{-(V-V_{0.5})}{k_a}}} \tag{A22}$$

$$\tau_{m_{\mathrm{EGL2}}}(V) = \frac{a}{1+e^{\frac{(V-b)}{c}}} + d \tag{A23}$$

$$I_{\mathrm{EGL2}} = \overline{g}_{\mathrm{EGL2}} \cdot m_{\mathrm{EGL2}} \cdot (V - V_K) \tag{A24}$$

EGL36

$$m^{\mathrm{f}}_{\mathrm{EGL36},\infty}(V) = m^{\mathrm{m}}_{\mathrm{EGL36},\infty}(V) = m^{\mathrm{s}}_{\mathrm{EGL36},\infty}(V) = \frac{1}{1+e^{\frac{-(V-V_{0.5})}{k_a}}} \tag{A25}$$

$$\tau^{\mathrm{f}}_{m_{\mathrm{EGL36}}} = \tau^{\mathrm{m}}_{m_{\mathrm{EGL36}}} = \tau^{\mathrm{s}}_{m_{\mathrm{EGL36}}} = a \tag{A26}$$

$$I_{\mathrm{EGL36}} = \overline{g}_{\mathrm{EGL36}} \cdot \left(0.33\, m^{\mathrm{f}}_{\mathrm{EGL36}} + 0.36\, m^{\mathrm{m}}_{\mathrm{EGL36}} + 0.39\, m^{\mathrm{s}}_{\mathrm{EGL36}}\right) \cdot (V - V_K) \tag{A27}$$

IRK

$$m_{\mathrm{IRK},\infty}(V) = \frac{1}{1+e^{\frac{(V-V_{0.5})}{k_a}}} \tag{A28}$$

$$\tau_{m_{\mathrm{IRK}}}(V) = \frac{a}{e^{\frac{-(V-b)}{c}} + e^{\frac{(V-d)}{\tilde{e}}}} + f \tag{A29}$$

$$I_{\mathrm{IRK}} = \overline{g}_{\mathrm{IRK}} \cdot m_{\mathrm{IRK}} \cdot (V - V_K) \tag{A30}$$

B. Voltage-gated calcium currents

EGL19

(B1) $$m_{\text{EGL19},\infty}(V) = \frac{1}{1 + e^{\frac{-(V - V_{0.5})}{k_a}}}$$

(B2) $$\tau_{m_{\text{EGL19}}}(V) = \left[a\ e^{-\left(\frac{V-b}{c}\right)^2}\right] + \left[d\ e^{-\left(\frac{V-\tilde{e}}{f}\right)^2}\right] + g$$

(B3) $$h_{\text{EGL19},\infty}(V) = \left[\frac{a}{1 + e^{\frac{-(V - V_{0.5})}{k_i}}} + b\right] \cdot \left[\frac{c}{1 + e^{\frac{\left(V - V_{0.5}^b\right)}{k_i^b}}} + d\right]$$

(B4) $$\tau_{h_{\text{EGL19}}}(V) = a\left[\frac{b}{1 + e^{\frac{(V-c)}{d}}} + \frac{\tilde{e}}{1 + e^{\frac{(V-f)}{g}}} + h\right]$$

(B5) $$I_{\text{EGL19}} = \overline{g}_{\text{EGL19}} \cdot m_{\text{EGL19}} \cdot h_{\text{EGL19}} \cdot (V - V_{Ca})$$

UNC2

(B6) $$m_{\text{UNC2},\infty}(V) = \frac{1}{1 + e^{\frac{-(V - V_{0.5})}{k_a}}}$$

(B7) $$\tau_{m_{\text{UNC2}}}(V) = \frac{a}{e^{\frac{-(V-b)}{c}} + e^{\frac{(V-b)}{d}}} + \tilde{e}$$

(B8) $$h_{\text{UNC2},\infty}(V) = \frac{1}{1 + e^{\frac{(V - V_{0.5})}{k_i}}}$$

(B9) $$\tau_{h_{\text{UNC2}}}(V) = \frac{a}{1 + e^{\frac{-(V-b)}{c}}} + \frac{d}{1 + e^{\frac{(V-\tilde{e})}{f}}}$$

(B10) $$I_{\text{UNC2}} = \overline{g}_{\text{UNC2}} \cdot m_{\text{UNC2}} \cdot h_{\text{UNC2}} \cdot (V - V_{Ca})$$

CCA1

(B11) $$m_{\text{CCA1},\infty}(V) = \frac{1}{1 + e^{\frac{-(V - V_{0.5})}{k_a}}}$$

(B12) $$h_{\text{CCA1},\infty}(V) = \frac{1}{1 + e^{\frac{(V - V_{0.5})}{k_i}}}$$

(B13) $$\tau_{m_{\text{CCA1}}}(V) = \frac{a}{1 + e^{\frac{-(V-b)}{c}}} + d$$

(B14) $$\tau_{h_{\text{CCA1}}}(V) = \frac{a}{1 + e^{\frac{(V-b)}{c}}} + d$$

(B15) $$I_{\text{CCA1}} = \overline{g}_{\text{CCA1}} \cdot m_{\text{CCA1}}^2 \cdot h_{\text{CCA1}} \cdot (V - V_{Ca})$$

C. Calcium-regulated potassium currents

SLO1-SLO2

CaV=EGL19, UNC2

$$m_{\mathrm{BK},\infty}(V,Ca)=\frac{m_{\mathrm{CaV}}k_o^+(\alpha+\beta+k_c^-)}{(k_o^++k_o^-)(k_c^-+\alpha)+\beta k_c^-} \tag{C1}$$

$$\tau_{m_{\mathrm{BK}}}(V,Ca)=\frac{\alpha+\beta+k_c^-}{(k_o^++k_o^-)(k_c^-+\alpha)+\beta k_c^-} \tag{C2}$$

$$\alpha=\frac{m_{\mathrm{CaV},\infty}}{\tau_{m_{\mathrm{CaV}}}} \tag{C3}$$

$$\beta=\tau_{m_{\mathrm{CaV}}}^{-1}-\alpha \tag{C4}$$

$$I_{\mathrm{BK}}=\overline{g}_{\mathrm{BK}}\cdot m_{\mathrm{BK}}\cdot h_{\mathrm{CaV}}\cdot(V-V_K) \tag{C5}$$

For k_o^+, k_o^-, k_c^- see Methods. Ca stands for the nano-scale calcium concentration close to CaV channels, i.e. $\left[\mathrm{Ca}^{2+}\right]_{o,i}^n$ and $\left[\mathrm{Ca}^{2+}\right]_{c,i}^n$ for $k_o^{+/-}$ and $k_c^{+/-}$, respectively.

KCNL

$$m_{\mathrm{KCNL},\infty}(Ca)=\frac{Ca}{K_{Ca}+Ca} \tag{C6}$$

$$\tau_{m_{\mathrm{KCNL}}}=a \tag{C7}$$

$$I_{\mathrm{KCNL}}=\overline{g}_{\mathrm{KCNL}}\cdot m_{\mathrm{KCNL}}\cdot(V-V_K) \tag{C8}$$

For intracellular calcium calculation see Methods. Ca stands for the micro-scale calcium concentration, i.e. the intracellular calcium $\left[\mathrm{Ca}^{2+}\right]_i^m$.

NCA

$$I_{\mathrm{NCA}}=\overline{g}_{\mathrm{NCA}}\cdot(V-V_{Na}) \tag{C9}$$

LEAK

$$I_{\mathrm{LEAK}}=\overline{g}_{\mathrm{LEAK}}\cdot(V-V_L) \tag{C10}$$
