# Supplementary figures and images for: Correction: Biophysical modeling of C. elegans neurons: Single ion currents and whole-cell dynamics of AWCon and RMD
Source: PLoS One. 2021 Aug 26;16(8):e0256930. doi: 10.1371/journal.pone.0256930 (PMC8389473; doi:10.1371/journal.pone.0256930)

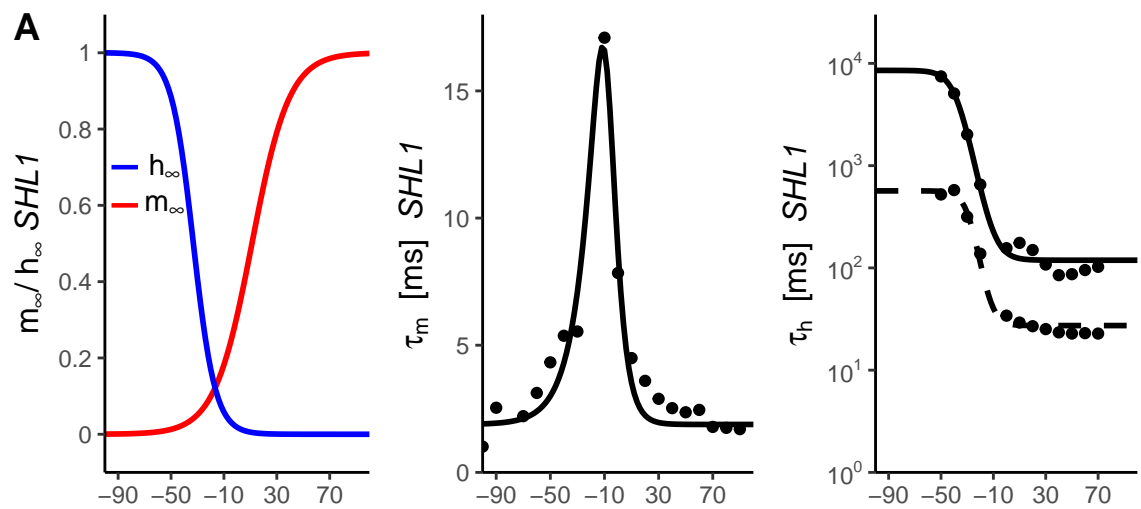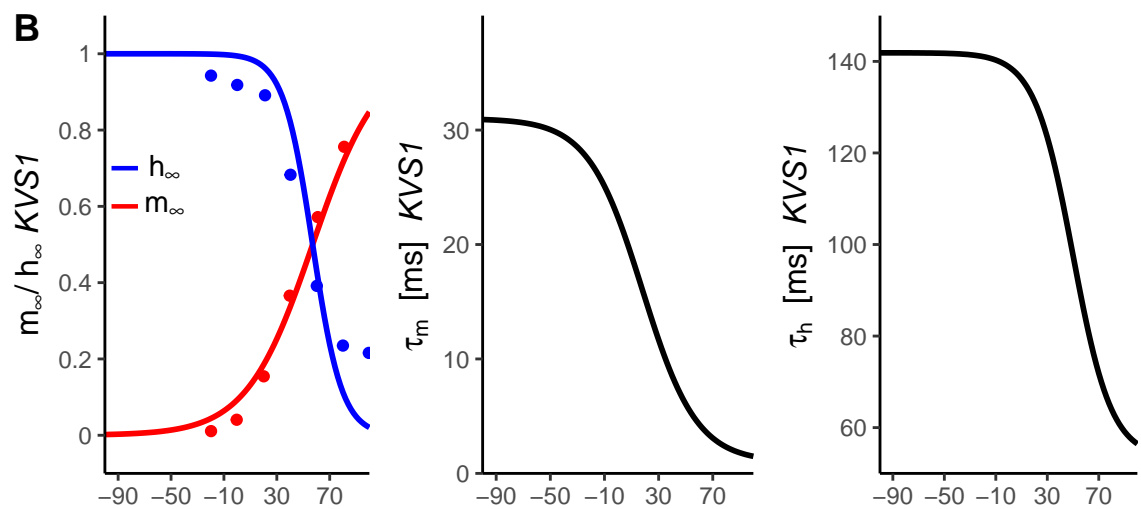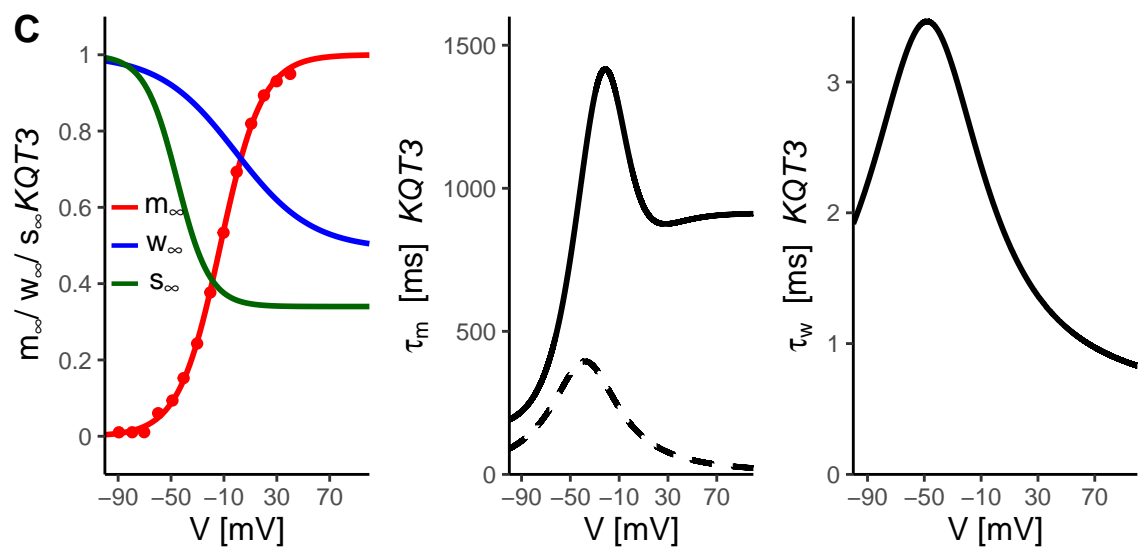

Supplement: S1 Fig — In panels A-C we report the steady-state activation and inactivation curves (left), the activation time constant function (center), and the inactivation time constant function (right). A) SHL1 currents. On the left we report steady-state activation (red) and inactivation (blue) functions (Eqs A1 and A3 in S1 File). In the middle the activation time constant function (τmSHL1S, Eq A2 in S1 File) is represented together with the fitted experimental values (black dots, from [25]). In the right panel the solid lines represent the slow inactivation time constant (τhSHL1S, Eq A4 in S1 File), while the dashed line describes the fast one (τhSHL1f, Eq A4 in S1 File). In this case, black dots are the experimental points extracted from [25]. B) KVS1 currents. Steady-state activation (red) and inactivation (blue), with experimental points (blue and red dots, from [31]), are represented on the left (Eqs A6 and A7 in S1 File). Middle and right panels show respectively activation and inactivation time constants as function of voltage (Eq A8 in S1 File, [31]). C) KQT3 currents. Steady-state activation (red, Eq A15 in S1 File, from [26]) and inactivation (blue and green, Eq A18 in S1 File, from [73]) variables are shown on the left. The red dots are experimental points from [26]. Middle panel shows fast (dashed) and slow (solid) activation time constants (Eqs A16 and A17 in S1 File, from [73]). On the left the inactivation time constant (τwKQT3, Eq A19 in S1 File) is represented. (PDF) [file pone.0256930.s003.pdf]

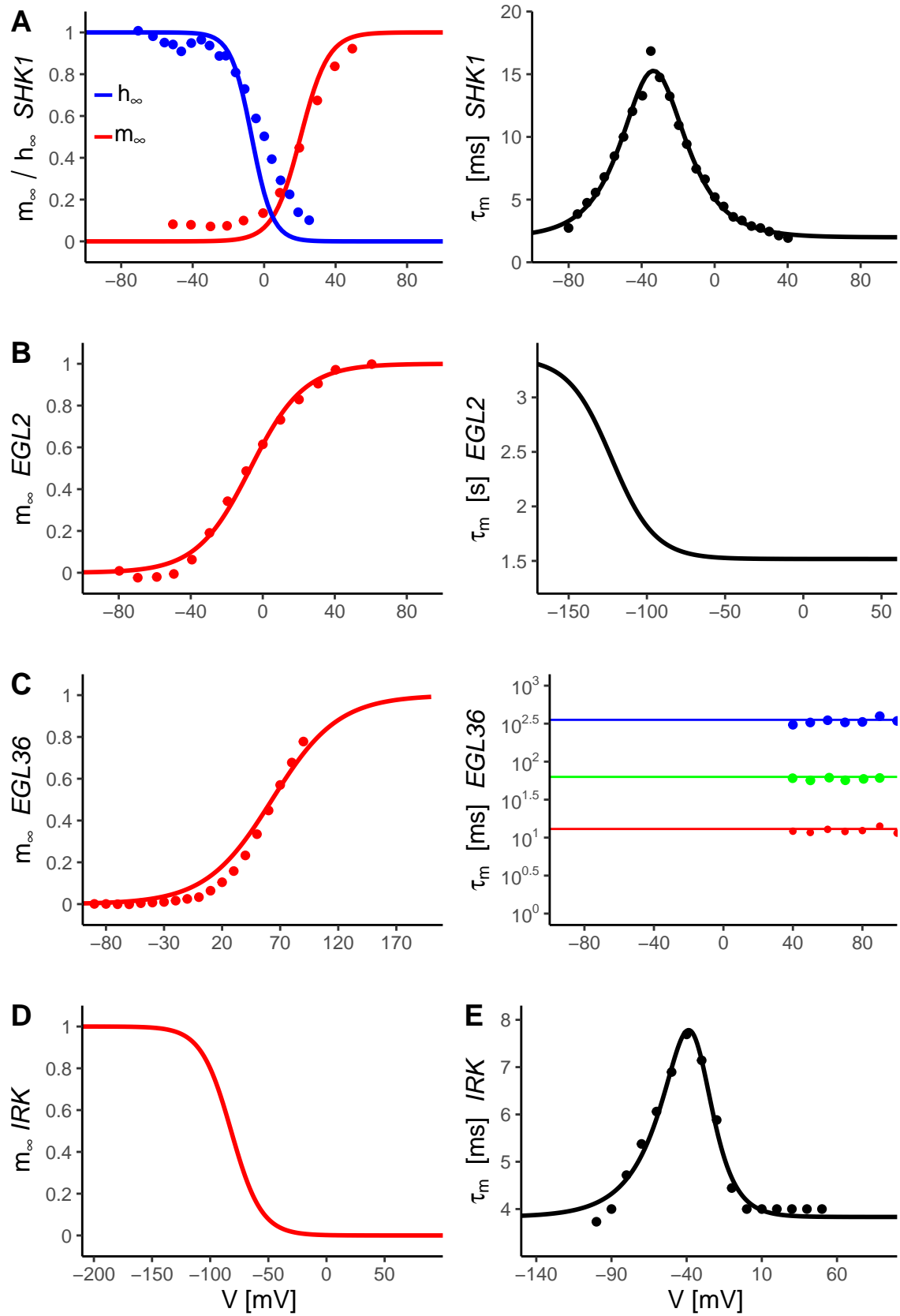

Supplement: S2 Fig — In panels A-D we report the steady-state activation and inactivation curves (left), and the activation time constant function (right). A) SHK1 currents. Steady-state activation (red, Eq A10 in S1 File) and inactivation (blue, Eq A12 in S1 File) functions are represented on the left. Blue and red dots are the experimental points from [25]. On the right the activation time constant function is shown (Eq A11 in S1 File) with fitted experimental points (black dots) from [29]. B) EGL2 currents. On the left is represented the steady-state activation variable (Eq A22 in S1 File) with experimental data (red dots) from [28]. On the left the activation time constant function (Eq A23 in S1 File) is shown. C) EGL36 currents. Steady-state activation variable (Eq A25 in S1 File) is shown on the left, with experimental data from [30] (red dots). Fast (red), medium (green) and slow (blue) activation time constants (Eq A26 in S1 File) are shown on the right. Red, green and blue dots represent experimental measurements as reported in [30]. D) IRK currents. Steady-state activation variable is represented on the right (Eq A28 in S1 File). On the left the activation time constant (Eq A29 in S1 File) is represented, with experimental data (black dots) from [35]. (PDF) [file pone.0256930.s004.pdf]
